# Effect of lipotoxic hepatocyte-derived extracellular vesicles in pancreas inflammation: essential role of macrophage TLR4 in beta cell functionality

**DOI:** 10.1007/s00125-025-06445-z

**Published:** 2025-05-19

**Authors:** Rosa Alén, Irma Garcia-Martinez, Nadia Cobo-Vuilleumier, Elisa Fernández-Millán, Paula Gallardo-Villanueva, Vitor Ferreira, Manuel Izquierdo, María Ángeles Moro, Ignacio Lizasoain, Natalia Nieto, Benoit R. Gauthier, Ángela M. Valverde

**Affiliations:** 1 Instituto de Investigaciones Biomédicas Sols-Morreale (IIBm, CSIC-UAM), Madrid, Spain; 2 Centro de Investigación Biomédica en Red de Diabetes y Enfermedades Metabólicas Asociadas (CIBERdem), Instituto de Salud Carlos III (ISCIII), Madrid, Spain; 3 Centro Andaluz de Biología Molecular y Medicina Regenerativa (CABIMER), Junta de Andalucía-University of Pablo de Olavide-University of Seville-CSIC, Sevilla, Spain; 4 Departamento de Bioquímica y Biología Molecular, Facultad de Farmacia, Universidad Complutense de Madrid, Madrid, Spain; 5 Fisiopatología Neurovascular, Centro Nacional de Investigaciones Cardiovasculares Carlos III (CNIC), Madrid, Spain; 6 Unidad de Investigación Neurovascular, Departamento de Farmacología y Toxicología, Facultad de Medicina, Universidad Complutense de Madrid, Madrid, Spain; 7 Instituto de Investigación Hospital 12 de Octubre (i+12), Madrid, Spain; 8 Department of Pathology, Department of Medicine (Gastroenterology and Hepatology), University of Illinois at Chicago, Chicago, IL USA

**Keywords:** Beta cells, Extracellular vesicles, Islet macrophages, Metabolic dysfunction-associated steatotic liver disease, Toll-like receptor 4, Type 2 diabetes

## Abstract

**Aims/hypothesis:**

Metabolic dysfunction-associated steatotic liver disease (MASLD) is a common feature of obesity and type 2 diabetes. Under lipotoxic stress, hepatocytes release small extracellular vesicles (sEVs) which act locally and contribute to MASLD progression, but their role in beta cell function and development of type 2 diabetes remains largely unexplored. We aimed to examine whether hepatocyte-derived sEVs (Hep-sEVs) under lipotoxic conditions impact on liver and pancreas inflammation and subsequent effects on beta cell function.

**Methods:**

Primary mouse hepatocytes and Huh7 human hepatocytes were treated with palmitic acid and Hep-sEVs were purified from the culture medium by differential ultracentrifugation. In vitro and in vivo approaches were used to decipher the role of Hep-sEVs in liver and pancreas inflammation and beta cell dysfunction in mouse and human pancreatic islets. The contribution of the Toll-like receptor 4 (TLR4) to Hep-sEV-mediated effects was investigated in pancreatic islets from myeloid-specific TLR4-deficient mice.

**Results:**

Lipotoxic Hep-sEVs targeted pancreatic islet macrophages and induced TLR4-mediated inflammation. The subsequent inflammatory response downregulated beta cell identity genes and impaired glucose-stimulated insulin secretion in both INS-1 beta cells (*p*<0.05) and isolated pancreatic islets from mice (*p*<0.01) and humans (*p*<0.05). Specific deletion of TLR4 in macrophages protected pancreatic islets against inflammation and the impairment of glucose-stimulated insulin secretion induced by lipotoxic Hep-sEVs. Chronic administration of lipotoxic Hep-sEVs in lean mice induced liver and pancreas inflammation through the recruitment of immune cells. This intervention induced hepatocyte injury and fibrotic damage together with detrimental immunometabolic systemic effects. Insulin resistance in hepatocytes (*p*<0.01) and a compensatory insulin secretion (*p*<0.001) that prevented glucose intolerance were also observed in mice treated with lipotoxic Hep-sEVs.

**Conclusions/interpretation:**

This study has provided evidence of liver and pancreas inflammation and beta cell dysfunction induced by lipotoxic Hep-sEVs. Our data also envision TLR4-mediated signalling in islet macrophages as a key mediator of the effects of lipotoxic Hep-sEVs on beta cell function.

**Graphical Abstract:**

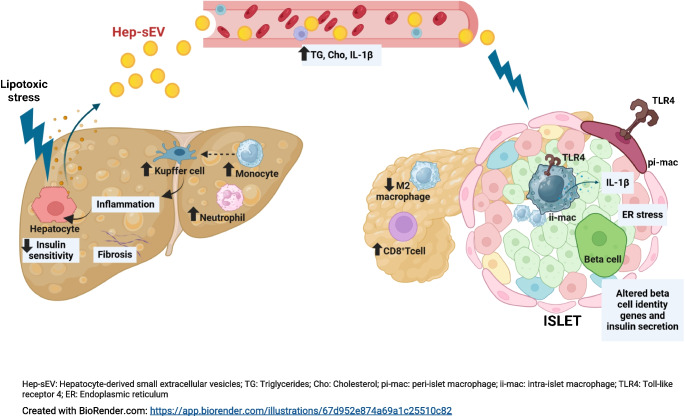

**Supplementary Information:**

The online version of this article (10.1007/s00125-025-06445-z) contains peer-reviewed but unedited supplementary material.


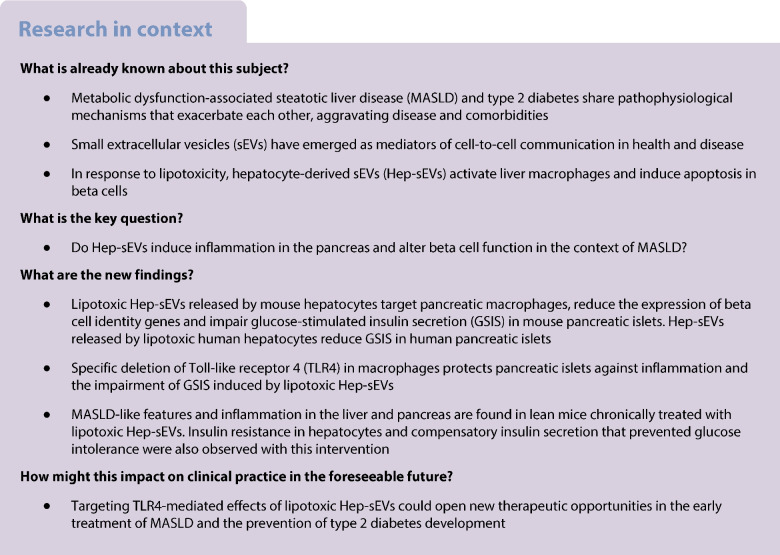


## Introduction

The association between type 2 diabetes and metabolic dysfunction-associated steatotic liver disease (MASLD), previously known as non-alcoholic fatty liver disease (NAFLD), is increasing in clinical practice, especially in Western countries. Both diseases have high individual prevalence and share metabolic risk factors, including genetic factors, lifestyle, obesity, dyslipidaemia and insulin resistance [1]. The prevalence of MASLD in individuals with type 2 diabetes is approximately 70–80%, whereas the prevalence of type 2 diabetes in individuals with MASLD, either obese or lean, is 2–3 times higher than in the general adult population [1, 2]. MASLD and type 2 diabetes act synergistically and, in most individuals, lead to more adverse hepatic and extra-hepatic outcomes. On one hand, MASLD exacerbates hepatic and peripheral insulin resistance which increases the risk of type 2 diabetes development and associated comorbidities and challenges its management. On the other hand, type 2 diabetes accelerates MASLD progression toward steatohepatitis (metabolic dysfunction-associated steatohepatitis [MASH]), cirrhosis and hepatocellular carcinoma [1]. Since improvement or resolution of MASLD is associated with reduction of type 2 diabetes risk, treatments targeting the liver might reduce type 2 diabetes development.

The liver has emerged as an endocrine organ which controls metabolic homeostasis by releasing hepatokines that target peripheral tissues including the pancreas. In fact, the liver–pancreas crosstalk is essential in glucose and lipid metabolism and in the development of insulin resistance and type 2 diabetes, with insulin and glucagon playing opposite roles [3, 4]. In this context, recent evidences point to the involvement of small extracellular vesicles (sEVs) released by steatotic hepatocytes in modulating beta cell proliferation [5] and apoptosis [6]. However, whether lipotoxic hepatocyte-derived sEVs (Hep-sEVs) during MASLD impair beta cell function, particularly insulin secretion, and the underlying molecular mechanisms have not been investigated.

Recently, we identified lipotoxic Hep-sEVs as transporters of saturated fatty acids (SFAs) and potent inducers of liver inflammation and insulin resistance in this organ by a mechanism dependent on macrophage Toll-like receptor 4 (TLR4) [7]. Herein, we have investigated the effects of chronic treatment of mice with lipotoxic Hep-sEVs in liver and pancreas inflammation, insulin action in hepatocytes and beta cell function, as well as the role of macrophages as drivers of the deleterious effects of lipotoxic Hep-sEVs on beta cells through TLR4-dependent inflammation.

## Methods

### Animals

Male mice on the C57BL/6J genetic background were maintained at the animal facilities of Instituto de Investigaciones Biomédicas Sols-Morreale (IIBm) (CSIC-UAM, Madrid, Spain). C57BL/6J male mice with global (TLR4^−/−^) or specific deletion of TLR4 in myeloid cells (TLR4^∆Mye^) and their respective controls (TLR4^+/+^ and TLR4^fl/fl^, respectively) were also used [8, 9]. Experiments were conducted in mice at 8–12 weeks of age. Animals were controlled following the recommendations of the Federation of European Laboratory Animal Science Associations (FELASA) on health monitoring. Mice were maintained in 12 h light/dark cycle, temperature (22°C) and humidity-controlled rooms, and fed a standard chow diet (8.4% energy from fat, A04, Panlab [Barcelona, Spain]). Mice were randomly assigned to the experimental groups. All experimental procedures were approved by the IIBm and CSIC Animal Care and Use Committees and authorised by the Comunidad de Madrid (Spain).

### Isolation and culture of primary hepatocytes

Primary hepatocytes were isolated from non-fasting C57BL/6J male mice by perfusion with collagenase following the classic two-step perfusion technique. See the electronic supplementary material (ESM) Methods for further details.

### Induction of sEV release by mouse primary hepatocytes

Primary hepatocytes from C57BL/6J male mice were seeded at equal confluence (2.5×10^6^ cells) in collagen-coated 150 mm dishes. At 24 h after plating, hepatocytes were cultured for a further 24 h in DMEM (5.5 mmol/l glucose) with 0.25% fatty acid-free BSA and without FBS in the absence or presence of palmitic acid (PA) (800 µmol/l). Thereafter, cell supernatants were collected for sEV isolation (sEV^C^ or sEV^PA^, respectively).

### Isolation of sEVs released by primary hepatocytes

sEVs were isolated by differential ultracentrifugation according to a modified protocol reported by Thery et al [10]. The culture supernatant of primary hepatocytes under the different experimental conditions was subjected to two low-speed centrifugation steps (300 × *g* for 12 min; 2000 × *g* for 12 min, 4°C) to remove cellular debris and apoptotic bodies. The supernatant was collected, filtered (0.22 μm) and ultracentrifuged at 10,000 × *g* and 100,000 × *g* at 4°C, for 35 min and 70 min, respectively, in a 60 Ti fixed-angle rotor. The pellet was resuspended in 1×PBS (10010-023, Gibco, Waltham, MA, USA) and ultracentrifuged again at 100,000 × *g* for 70 min at 4°C in a TLA-100 fixed-angle rotor (Beckman-Coulter, Brea, CA, USA). This procedure ensures uniformity of purified vesicles in the resulting pellet and prevents contamination by medium/large extracellular vesicles (EVs) (size >200 nm) or apoptotic bodies (1–5 μm). Each final pellet of sEVs (size <100 nm or <200 nm), isolated from equal numbers of primary hepatocytes, was resuspended in an equal volume of PBS. The characterisation of sEVs released by primary mouse hepatocytes was previously described [7].

### Staining of sEVs and in vivo biodistribution

To evaluate sEV biodistribution in the pancreas, 50 µg of Hep-sEVs, determined by BCA assay (23227, ThermoFisher, USA), were labelled with PKH26 (red) (MINI26-1KT, Sigma-Aldrich, USA). Staining was performed according to the manufacturer’s protocol with minor modifications. Stained sEVs were washed five times with 100 kDa Vivaspin filters (VS0141, Sartorius, Göttingen, Germany) to remove excess dye in downstream applications. C57BL/6J male mice were injected with sEVs via tail vein and culled after 24 h. Pancreatic tissue was fixed with 4% paraformaldehyde (PFA) (16005, Sigma-Aldrich, San Louis, MO, USA) overnight and sEV biodistribution was monitored by fluorescence immunohistochemistry.

### In vitro treatment of macrophages, INS-1 beta cells and mouse islets with sEVs

The sEVs purified from the hepatocyte culture medium were resuspended in 50 μl of PBS for each hepatocyte dish (2.5×10^6^ cells) and the 50 μl of resuspended sEVs (1–5×10^10^ sEVs determined by nanoparticle tracking analysis [NTA]) were added to each well of peritoneal macrophages (1.25×10^6^ cells), INS-1 beta cells (4×10^5^ cells) or mouse islets (50 islets). In some experiments Hep-sEVs were labelled with PKH26 to monitor their uptake by INS-1 cells, peritoneal macrophages and mouse pancreatic islets.

### Isolation, culture and generation of conditioned medium from peritoneal macrophages

Thioglycolate 3% (wt/vol.) (225650, BD, Franklin Lakes, NJ, USA) was peritoneally injected into C57BL/6J or TLR4^−/−^ male mice 3–4 days prior to isolation to increase macrophage yield. See the ESM Methods for further details.

### Culture of and studies in INS-1 beta cell line

Mycoplasma-free rat insulinoma INS-1 cells were cultured in RPMI medium containing 11 mmol/l glucose supplemented with 10% heat-inactivated FBS, 10 mmol/l HEPES, 2 mmol/l glutamine, 1 mmol/l sodium pyruvate, 100 U/ml penicillin, 100 µg/ml streptomycin and 50 μmol/l β-mercaptoethanol. INS-1 cells were seeded at a density of 3×10^5^ cells/well on 12-well plates and maintained in INS-1 medium for 24 h before treatment. Experiments were performed in INS-1 medium supplemented with 10% EV-depleted FBS.

INS-1 beta cells were incubated directly with Hep-sEVs or with conditioned medium (CM) collected from Hep-sEV-treated peritoneal mouse macrophages for 24 h. To determine cell viability, INS-1 cells were fixed with 4% PFA for 10 min. Then, the cells were stained with crystal violet (0.1% wt/vol.) for 30 min. After this time, the plates were rinsed with tap water and allowed to dry and, finally, 10% (vol./vol.) acetic acid was added to allow solubilisation. The absorbance of each plate was read in a spectrophotometer at 590 nm. For the analysis of apoptotic nuclei, INS-1 cells were washed twice with 1×PBS, fixed in 4% PFA for 10 min and stained with DAPI (D1306, Molecular Probes).

Endoplasmic reticulum (ER) stress and inflammation pathways were examined at the time points indicated in the corresponding figures by western blot with primary antibodies against p-eIF2α (eukaryotic translation initiation factor 2α), eIF2α, p-JNK (c-Jun N-terminal kinase), JNK, IκBα (NF-κB inhibitor α) and caspase 1. At 1 h post treatment with sEVs, p65-NF-κB nuclear translocation was also evaluated by cell immunofluorescence. At 6 h post treatment with sEVs, expression levels of ER stress markers (*Ddit3* mRNA levels and *Xbp-1* splicing), inflammatory markers (*Il1b* mRNA levels) and beta cell genes (*Pdx1*, *Ins1*, *Ins2* mRNA) were assessed by reverse transcription real-time quantitative PCR (RT-qPCR).

At 6 or 24 h post treatment with CM or sEVs, respectively, glucose-stimulated insulin secretion (GSIS) capacity and insulin content were analysed by ELISA (10-1247-01, Mercodia, Uppsala, Sweden) following the manufacturer’s instructions. INS-1 cells were pre-incubated for 1 h at 37°C in KRB solution containing 2.8 mmol/l glucose. Afterwards, the KRB solution was replaced with 0.5 ml of fresh KRB solution supplemented with 2.8 mmol/l glucose, and INS-1 cells were then incubated for 1 h at 37°C. The supernatant was collected into a 1.5 ml tube and cells were incubated again with 0.5 ml of KRB solution supplemented with 16.7 mmol/l glucose for 1 h at 37°C. The supernatant was also collected into a 1.5 ml tube. Both tubes were centrifuged at 5600 × *g* for 10 min at 4°C, and the supernatants were collected and stored at −20°C until analysis. INS-1 cells were lysed in glycine/NP-40 lysis buffer (200 mmol/l glycine, 0.5% NP-40; pH 8.8) and the samples were centrifuged for 15 min at 16,000 × *g* and the supernatants stored at −20°C until analysis of insulin content by ELISA. Values were normalised to protein content.

### Isolation and culture of mouse pancreatic islets

Pancreatic islets were isolated from non-fasting C57BL6J male mice by in situ pancreas perfusion, enzymatic digestion and several sedimentation steps. Per mouse, 3 mg of collagenase (S1745601, Nordmark Pharma, Uetersen, Germany) was dissolved in 5 ml of Hanks' Balanced Salt Solution (HBSS) solution. Following mouse euthanasia, the common bile duct and hepatic artery bundle near the liver end were clamped. Each side of the ampulla of Vater in the intestine was also clamped to avoid leakage. Then, 3 ml of collagenase solution was slowly and steadily injected via the ampulla of Vater using a 30 gauge needle. This method uses the network of ducts present in the pancreas to introduce the collagenase. The remaining 2 ml of collagenase solution was placed in a 50 ml tube together with the perfused pancreas and incubated in a shaking water bath at 37°C for 20–25 min to expedite the digestion mechanically. Enzymatic digestion was stopped by adding cold HBSS solution. After several washing, shaking and sedimentation steps with HBSS solution, pancreatic islets were hand-picked and cultured in islet culture medium for 24 h before treatment. Experiments were performed in islet culture medium supplemented with 10% EV-depleted FBS.

### Ex vivo assays in pancreatic islets

Pancreatic islets from C57BL/6J mice were treated ex vivo with Hep-sEVs for 1 or 2 h to examine p65-NF-κB nuclear translocation in islet macrophages using an anti-F4/80 primary antibody by in toto immunostaining.

Hep-sEVs were also added to pancreatic islets to study beta cell gene expression (*Pdx1*, *Ins1*, *Ins2*, *Gck*) and proinflammatory markers (*Il1b*, *Adgre1*, *Tlr4*) by RT-qPCR.

CM from pancreatic islets treated with Hep-sEVs for 48 h was collected and immediately frozen at −80°C. IL-1β concentration was determined using ELISA (BMS6002, Invitrogen, Waltham, MA, USA).

For experiments with clodronate, islets were isolated and maintained for 4 h in complete medium and then macrophages were depleted using clodronate-loaded liposomes (1 mg/ml, Liposoma, the Netherlands) for 48 h. After that, islets were washed carefully with 1% BSA in PBS and then stimulations with sEVs were conducted.

GSIS test was conducted at 24 h post treatment with sEVs. For each mouse, 4–8 groups of three islets matched by size were placed in each well of a 96-well plate. Islets were pre-incubated for 1 h at 37°C in KRB solution containing 2.8 mmol/l glucose. Incubations were then performed using 2.8 mmol/l or 16.7 mmol/l glucose at 37°C for 1 h. Insulin was extracted from 20–24 islets per mouse using glycine/NP-40 lysis buffer (200 mmol/l glycine, 0.5% NP-40; pH 8.8). Insulin levels and insulin content were determined by ELISA (10-1247-01, Mercodia, Sweden) and normalised to islet number.

### Chronic injection of sEVs in lean mice

Hep-sEVs released from equal numbers of primary hepatocytes (2.5×10^6^) isolated from C57BL/6J male mice, with or without PA treatment (sEV^C^ and sEV^PA^), were injected through the tail vein into lean C57BL/6J male mice twice a week for 4 weeks. ITT, GTT and GSIS were performed 24 h before the last injection. Mice were culled 24 h after the last sEV injection. Blood was collected to measure different biochemical parameters. The liver and pancreas were collected for isolation of immune cell subpopulations and immunohistochemistry analysis. Another sub-cohort of treated mice was perfused to study ex vivo insulin signalling and glucose production in primary hepatocytes and GSIS in pancreatic islets.

### Haematoxylin and eosin staining in liver sections

Paraffin-embedded liver sections were deparaffinised and re-hydrated, immersed in haematoxylin for 1 min, then in eosin for 2 min and placed under running tap water until clear. Slides were then de-hydrated, cleared with xylene and mounted in DePex. Random fields at ×20 magnification were evaluated using a Zeiss AxioPhot microscope (Carl Zeiss, Oberkochen, Germany).

### Sirius red staining in liver sections

Paraffin-embedded liver sections were deparaffinised and re-hydrated, then immersed for 1 h in 0.1% Sirius red in saturated picric acid (1.3% in water). Slides were washed two times in acidified water (0.5% glacial acetic acid), de-hydrated in three changes of 100% ethanol, cleared with xylene and mounted in DePex. Random fields at ×20 magnification were evaluated using a Zeiss AxioPhot microscope (Carl Zeiss).

### Isolation of hepatic non-parenchymal cells

Mouse livers were collected in wash buffer (1% BSA in PBS) and gently pushed through a 70 µm cell strainer into a 50 ml tube. Then, 5 ml of Percoll was added to the homogenate, vortexed and centrifuged at 500 × *g* for 15 min at room temperature. The pellet was resuspended in wash buffer and centrifuged again at 600 × *g* for 6 min at 4°C. The pellet was resuspended in ACK buffer (50 mmol/l NH_4_Cl, 10 mmol/l KHCO_3_, 0.1 mmol/l EDTA pH 7.3) after which a 5 min buffer wash was performed. The suspension was centrifuged at 600 × *g* for 6 min at 4°C. The resulting cell pellet enriched in non-parenchymal cells (NPCs) was resuspended in 1 ml of heat-inactivated FBS with 10% DMSO (D8418, Sigma-Aldrich, USA) and stored in liquid nitrogen until analysis.

### Isolation of pancreatic immune cell subpopulations

Pancreas was detached from fat, pancreatic lymph nodes and spleen, and collected in a Petri dish containing cold 1×HBSS with 10% heat-inactivated FBS. The pancreas was cut by mincing and then transferred to a 50 ml tube. Once the tissue was sedimented, it was resuspended in HBSS–10% FBS with collagenase (S1745601, Nordmark, Germany) (20 mg/mouse) and incubated for 15 min at 37°C, shaking vigorously every 5 min. The digested pancreas was filtered through a 70 µm cell strainer and centrifuged at 500 × *g* for 5 min at 4°C. The pellet was resuspended in ACK buffer and after 5 min HBSS–10% FBS was added. The suspension was centrifuged at 500 × *g* for 5 min at 4°C. The resulting cell pellet enriched in immune cells was resuspended in 1 ml of heat-inactivated FBS with 10% DMSO and stored in nitrogen until analysis.

### Flow cytometry analysis of immune cells

Immune cells extracted from mouse liver and pancreas stored in nitrogen were thawed in RPMI medium supplemented with 10% heat-inactivated FBS and centrifuged at 300 × *g* for 5 min at 10°C. The pellet was resuspended in staining solution (PBS, 5% FBS, 2 mmol/l EDTA). Additionally, immune cells from the pancreas were filtered through a 70 µm cell strainer. Cells were counted and centrifuged at 300 × *g* for 5 min at 10°C. Then, cells were incubated with 100 μl of an Fc receptor blocker (1:100) (1630-01, Southern Biotech, Birmingham, AL, USA) for 10 min on ice. This step reduces non-specific binding and background fluorescence from Fc receptors. Cells were resuspended in staining solution, split into cytometry tubes for labelling and centrifuged at 300 × *g* for 5 min at 10°C. For cell surface staining, surface primary antibodies (ESM Table 1) were incubated for 30 min at 4°C in the dark, washed in staining solution and centrifuged at 300 × *g* for 5 min at 10°C. The resulting pellet was resuspended in 300 μl of staining solution for flow cytometry analysis. For cell death discrimination, cells were incubated with 7-aminoactinomicin D (7AAD) (00-6993-50, ThermoFisher, Waltham, MA, USA) for 3 min at room temperature.

Immune cells were acquired with a CytoFLEX S (Beckman Coulter, Brea, CA, USA). Cells were gated based on forward and side scatter. The square delineated subpopulation was then used to characterise the specific populations using the following antibodies: CD45, F4/80, CD11b, CD206 for macrophages; CD45, CD11b and Ly6c/Ly6g for monocytes or neutrophils; and CD45, CD3 and CD4/CD8 for helper or cytotoxic T cells. Data were analysed using CytExpert 2.4 software (Beckman Coulter).

### Biochemical analysis

Alanine aminotransferase (ALT) activity was determined spectrophotometrically in mouse plasma samples using a modified kinetic method of the International Federation of Clinical Chemistry and Laboratory Medicine (IFCC) (Pointe Scientific). ALT activity was calculated based on the mean absorbance difference/min (∆absorbance/min × 16,238). Triglycerides (TG) and cholesterol were analysed in liver and plasma samples using commercial kits (Pointe Scientific) as indicated by the manufacturer. For liver samples, 50 mg of tissue was homogenised in 500 μl of PBS and measured spectrophotometrically. Liver TG and cholesterol levels were normalised to protein concentration. NEFAs were quantified spectrophotometrically with the NEFA-HR Assay (91775, Wako Chemicals, Neuss, Germany).

### Insulin signalling and glucose production by primary hepatocytes

Insulin signalling and glucose production were analysed in primary hepatocytes from mice receiving chronic treatment with sEVs. See the ESM Methods for further details.

### Metabolic assays

Before the last sEV injection in the chronic treatment in mice, ITT, GTT and GSIS tests were performed in the experimental groups of mice. In the ITT, insulin (0.75 U/kg body weight; Actrapid, Novo Nordisk) was injected intraperitoneally in mice after 4 h of fasting. Plasma glucose levels were measured at 0, 15, 30, 60, 90 and 120 min post insulin injection with a glucometer (Accu-Check Aviva, Roche Diagnostics, Switzerland). In the GTT, d-(+)-Glucose (2 g/kg body weight; G8270, Sigma-Aldrich, USA) was injected intraperitoneally in mice after 16 h of fasting. Plasma glucose levels were measured at 0, 15, 30, 60, 90 and 120 min post glucose injection with a glucometer (Accu-Check Aviva, Roche Diagnostics). In the GSIS test, glucose (3 g/kg) was injected intraperitoneally in mice after 16 h of fasting. Blood samples were collected from the tail vein at 0, 3, 10 and 30 min and insulin levels were measured with a mouse insulin ELISA kit (10-1247-01, Mercodia, Sweden).

### Analysis of beta cell area

To avoid any bias due to regional changes in islet distribution and islet cell composition, each paraffin block (including the whole pancreas) was serially sectioned (5 µm) using a microtome (Leica RM2125RT) along its entire length and mounted on glass slides. Immunostaining was then performed on sections at fixed intervals throughout the block. Double immunostaining against insulin and glucagon was performed in order to study islet integrity and to quantify beta cell fractional area. Sections were blocked with goat serum (S-1000, Vector Laboratories) and incubated with primary mouse antibody against glucagon and guinea pig anti-insulin, overnight at 4°C. Subsequently, secondary goat anti-mouse antibody conjugated with peroxidase (A4416, Sigma-Aldrich) or conjugated with alkaline phosphatase (106-056-003, Jackson ImmunoResearch, West Grove, PA, USA) was added, and the sections were finally developed with a 3,3′-diaminobenzidine (DAB) or alkaline substrate kit (SK-4100 or SK-5100, respectively, Palex Medical, Barcelona, Spain) and counterstained with Harris's haematoxylin. Images of the sections were acquired using a digital camera connected to a Nikon Eclipse 80i microscope. The percentage of beta cell area was analysed using ImageJ v1.8 software (NIH) and expressed relative to the total pancreatic area measured in the sections of each condition. At least 30 sections per condition were analysed.

### Culture of Huh7 human hepatocyte cell line

Human hepatocellular carcinoma Huh7 cells (mycoplasma-free) were grown in DMEM supplemented with 10% heat-inactivated FBS, 100 U/ml penicillin and 100 μg/ml streptomycin. Cells were seeded on 12-well plates and maintained for 24 h before treatment. Treatments were conducted in DMEM supplemented with 1% EV-depleted FBS, 0.25% BSA and antibiotics.

### Induction of sEV release by Huh7 hepatocytes and characterisation

Huh7 hepatic cells were seeded on 12-well plates and maintained for 24 h in DMEM supplemented with 10% heat-inactivated FBS, 100 U/ml penicillin and 100 μg/ml streptomycin and treated with 1% EV-depleted FBS, 0.25% BSA and antibiotics in the absence or presence of 200 µmol/l PA for 24 h to allow sEV release.

sEVs isolated from Huh7 hepatic cells were characterised by western blot with antibodies against the sEV-associated proteins: anti-CD63 (non-reducing conditions), anti-CD81 and anti-tumour susceptibility gene 101 (TSG101). Anti-proliferating cell nuclear antigen (PCNA) and anti-glucose regulated protein 78 (GRP78) antibodies were used as negative controls. The concentration of sEVs (particles/ml) was determined by NTA with Nanosight LM10 equipment (Malvern Panalytical, Malvern, UK). The system was equipped with a fast video-capture and particle-tracking software. For each sample, at least four videos of 60 s with more than 200 detected tracks per video were taken and analysed using the Nanosight NTA 2.3 software (Malvern Panalytical, UK) with camera level 13 and detection threshold 3 settings. Results are represented as mean concentration (±SD) of sEVs that was obtained from the analysis of the four videos.

### Human pancreatic islets: culture, treatment with sEVs and GSIS

Human pancreatic islets from three donors were obtained from Tebubio (Le Perray-en-Yvelines, France). The Human islets checklist is included in the ESM. Islet preparations were washed, hand-picked and subsequently maintained in CMRL-1066 (P04-84600, Pan-Biotech, Germany) containing 5.5 mmol/l glucose supplemented with 0.5% BSA, 100 U/ml penicillin, 100 μg /ml streptomycin, 1% glutamine and 1% ITS (complete medium), for 16–24 h before treatment with sEVs. Experiments for each donor were performed with a different batch of sEVs isolated from Huh7 human hepatocytes. After washing and hand-picked selected, 100–200 islets from each donor were available for experiments. Islets were plated in non-adherent 12-well plates in groups of 50 islets/well and stimulated with sEV^C^ or sEV^PA^ (10^10^ sEVs determined by NTA) for 24 h and then GSIS was conducted. Islets were pre-incubated for 1 h at 37°C/5% CO_2_ in KRBH solution containing 2.8 mmol/l glucose. Next, islets for each condition were separated in Eppendorf tubes (low binding) into groups of 12 by triplicate and incubated in KRBH solution containing 2.8 mmol/l glucose at 37°C/5% CO_2_ (with lids open) for 1 h. Then, islets were carefully centrifuged and supernatants were collected. After that, islets were incubated in KRBH containing 16.7 mmol/l glucose at 37°C/5% CO_2_ (with lids open) for 1 h. Islets were carefully centrifuged and supernatants were collected. Insulin was extracted from 12 islets/condition using 500 µl of acid ethanol solution (95% ethanol, acetic acid, HCl). Insulin levels and content were determined by ELISA (10-1113-01, Mercodia, Sweden) and values were normalised to islet number.

### Immunostaining procedures

Fluorescence immunocytochemistry in INS-1 cells, in toto immunofluorescence of pancreatic islets, fluorescence immunohistochemistry in liver and pancreatic tissue and TUNEL assay in pancreatic sections were conducted. Antibodies for immunostaining procedures are listed in ESM Table 2. See the ESM Methods for further details.

### RT-qPCR

RT-qPCR was used to determine the relative expression levels of mRNAs. Primer sequences and TaqMan probes used are shown in ESM Table 3. See the ESM Methods for further details.

### Protein extracts preparation and western blot

Protein expression levels in INS-1 cells, primary mouse hepatocytes and sEVs released by hepatocytes were determined. Antibodies for western blot are listed in ESM Table 4. See the ESM Methods for further details.

### Statistical analysis

Statistical analysis was performed using GraphPad Prism 8.4.2 software (San Diego, CA, USA). Statistical details are provided in each figure legend. Differences between two groups were compared using Mann–Whitney *U* test. In ex vivo experiments GSIS was analysed by two-way ANOVA, followed by Bonferroni post hoc test. GTT and GSIS in vivo were analysed by one-way ANOVA, followed by Bonferroni post hoc test. Data are expressed as the mean±SEM. A *p* value of less than 0.05 was considered significant. Mice and cells were randomly and blindly distributed for the treatments by investigators. Investigators were not blind in outcome assessment.

## Results

### Lipotoxic Hep-sEVs target islet macrophages and induce a proinflammatory response in beta cells resulting in impaired insulin secretion

**Fig. 1:**
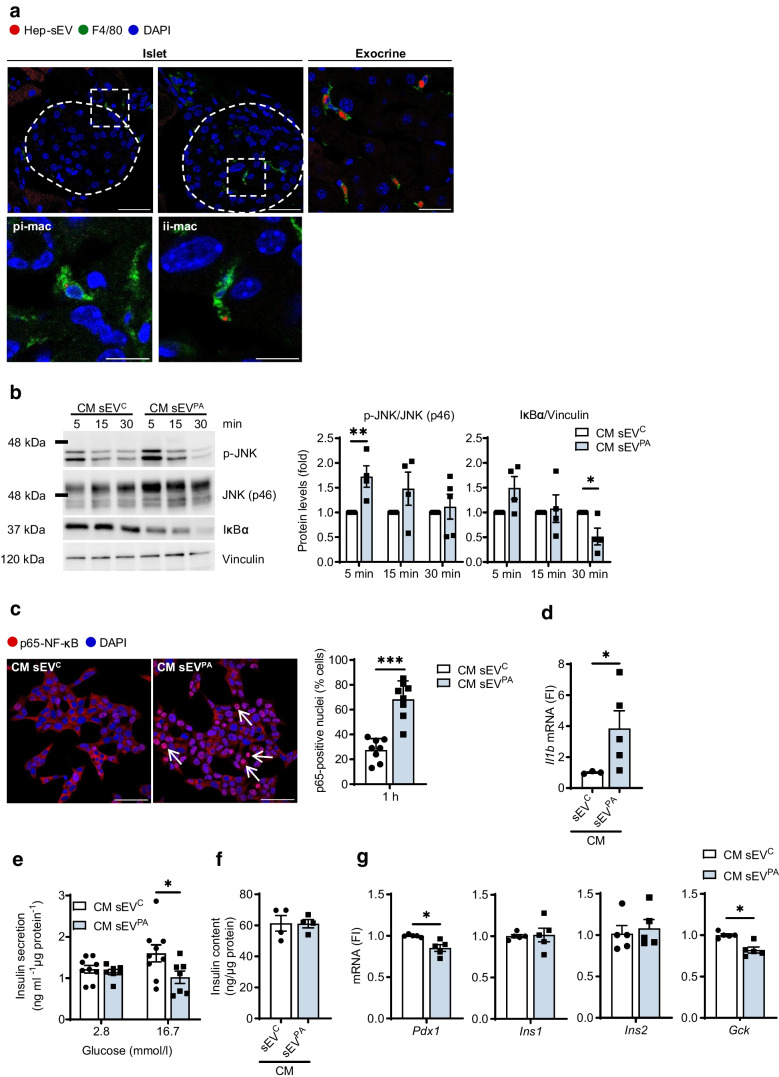
Effect of the CM from peritoneal macrophages treated with Hep-sEVs in INS-1 cells. (**a**) PKH26-labelled sEV^PA^ uptake by pi-macs and ii-macs and macrophages on pancreatic exocrine tissue. Representative images of pancreas (scale bars, 30 µm in upper panels and 10 µm in lower panels) are shown. The dotted white line depicts the border of an islet. (**b**) Representative western blot analysis with the indicated antibodies: p-JNK/JNK (p46) (*n*=4–5/group) and IκBα (*n*=4/group) and quantification, plotted as fold relative to CM sEV^c^ at each time point. (**c**) p65-NF-κB nuclear translocation by immunofluorescence (red) counterstaining with DAPI (blue). Arrows point to p65-NF-κB-positive nuclei (scale bar, 50 µm). Quantification of the percentage of cells with p65-NF-κB nuclear translocation (*n*=8/group). (**d**) *Il1b* mRNA levels at 6 h (sEV^C^ *n*=3, sEV^PA^ *n*=5). (**e**) GSIS (ng ml^−1^ µg protein^−1^) (sEV^C^ *n*=9, sEV^PA^ *n*=7), (**f**) insulin content (ng/µg protein) (*n*=4/group) and (**g**) beta cell identity gene expression (*n*=5/group) at 6 h. Data are expressed as the mean ± SEM. In (**b**, **c**, **d** and **g**): *p<0.05, **p<0.01, ***p<0.001, compared with CM-sEV^C^, Mann–Whitney *U* test. In (**e**): *p<0.05, compared with sEV^C^ at 16.7 mmol/l glucose, two-way ANOVA and Bonferroni post hoc test. FI, fold induction

We have recently reported that lipotoxic Hep-sEVs rapidly target liver macrophages upon i.v. injection in lean mice [7]. To decipher possible effects of lipotoxic Hep-sEVs in the pancreas, we first analysed their in vivo biodistribution in this tissue. To achieve this, Hep-sEVs were isolated from equal numbers of mouse primary hepatocytes treated for 24 h with 800 μmol/l PA, an SFA that mimics lipotoxicity in vitro, or BSA as control. As shown in Fig. 1a, PKH26-labelled sEVs were detected at 24 h post injection in F4/80-positive macrophages both in the peripheral islet area (peri-islet macrophages [pi-macs]) and within the islet (intra-islet macrophages [ii-macs]), as well as in the exocrine pancreatic tissue. As we found that Hep-sEVs target macrophages in pancreatic islets, we first conducted in vitro experiments in INS-1 beta cells to analyse the crosstalk between macrophages and beta cells mediated by Hep-sEVs. Incubation of INS-1 cells with the CM collected from macrophages treated with sEV^C^ (CM-sEV^C^) or sEV^PA^ (CM-sEV^PA^) did not alter cellular viability (ESM Fig 1a). CM-sEV^PA^, enriched in IL-1β and IL-6 as we previously reported [7], activated NF-κB-mediated proinflammatory signalling, as shown by IκBα degradation, as well as JNK phosphorylation in INS-1 beta cells (Fig. 1b). Of note, the CM-sEV^C^ did not affect these responses (Fig. 1b, ESM Fig. 2a). CM-sEV^PA^ also induced p65-NF-κB nuclear translocation (Fig. 1c) and elevation of *Il1b* mRNA (Fig. 1d) in INS-1 beta cells. Moreover, GSIS was decreased in INS-1 cells receiving CM-sEV^PA^ compared with cells treated with CM-sEV^C^ (*p*<0.05) (Fig. 1e) without differences in insulin content (Fig. 1f). This effect was accompanied by a reduction in *Pdx1* and *Gck* mRNAs in INS-1 cells receiving CM-sEV^PA^ (Fig. 1g) without changes in nuclear pancreatic and duodenal homeobox 1 (PDX1) localisation (ESM Fig. 3a).

### Lipotoxic Hep-sEV-mediated NF-κB activation in mouse pancreatic islets is driven by islet macrophages and impairs insulin secretion

**Fig. 2:**
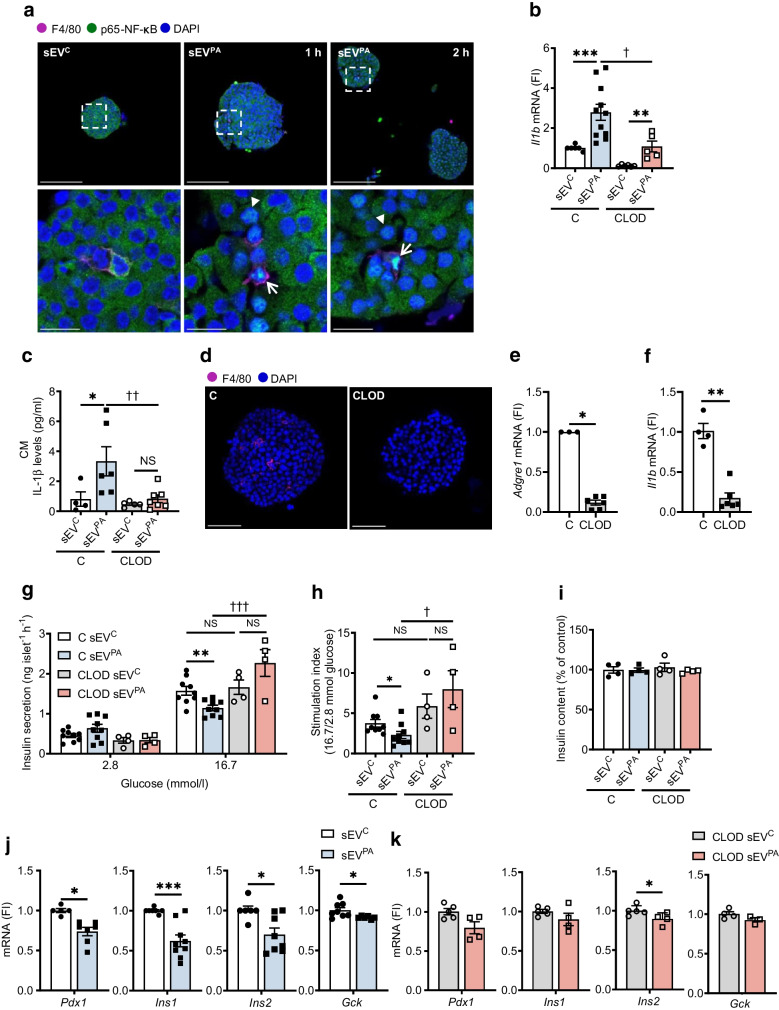
Effect of Hep-sEVs in control and CLOD islets ex vivo. (**a**) p65-NF-κB nuclear translocation by immunofluorescence (green) counterstaining with DAPI (blue) in pancreatic islets at 1 and 2 h post sEV addition. p65-NF-κB-positive nuclei in ii-macs (F4/80 magenta staining, arrow) and beta cells (arrowhead). Scale bar, 100 µm (upper panels) or 15 µm (lower panels). (**b**) *Il1b* mRNA levels in control (C) and CLOD islets 8 h post addition of Hep-sEVs (C sEV^C^ *n*=6, C sEV^PA^ *n*=11, CLOD sEV^C^ *n*=6, CLOD sEV^PA^ *n*=5). (**c**) IL-1β released to the culture supernatants by C and CLOD islets treated with Hep-sEVs for 48 h (C sEV^C^ *n*=4, C sEV^PA^ *n*=6, CLOD sEV^C^ *n*=5, CLOD sEV^PA^ *n*=7). (**d**) F4/80 staining (magenta) and counterstaining with DAPI (blue) in C and CLOD islets (scale bar, 50 µm). (**e**) *Adgre1* and (**f**) *Il1b* mRNA levels in C and CLOD islets (in **e**: C *n*=3, CLOD *n*=6; in **f**: C *n*=4, CLOD *n*=6). (**g**, **h**) GSIS (ng islet^−1^ h^−1^) by C and CLOD islets treated with Hep-sEVs for 24 h (C *n*=9/group, CLOD *n*=4/group). Experiments were performed with eight technical replicates per mouse/condition; panel (**h**) shows the stimulation index. (**i**) Insulin content (% of control) in C and CLOD islets treated with Hep-sEVs for 24 h (*n*=4/group). (**j**) Beta cell identity gene expression in C islets treated with Hep-sEVs for 8 h (*n*=5–9/group). (**k**) Beta cell identity gene expression in CLOD islets treated with Hep-sEVs for 8 h (*n*=3–5/group). Data are expressed as the mean ± SEM. In (**b** and **c**): *p<0.05, ***p<0.001, compared with sEV^C^ from the same condition, Mann–Whitney *U* test; ^†^*p*<0.05, ^††^*p*<0.01, compared with sEV^PA^ in C islets, Mann–Whitney *U* test. In (**e** and **f**): *p<0.05, **p<0.01, compared with C, Mann–Whitney *U* test. In (**g**): **p<0.01, compared with sEV^C^ at 16.7 mmol/l glucose from the same condition, two-way ANOVA and Bonferroni post hoc test; ^†††^*p*<0.001, compared with sEV^PA^ at 16.7 mmol/l glucose in C islets, two-way ANOVA and Bonferroni post hoc test. In (**h**): ^†^*p*<0.05, compared with sEV^PA^ in C islets; *p<0.05, compared with sEV^C^ in the same condition, Mann–Whitney *U* test. In (**j** and **k**): *p<0.05, ***p<0.001, compared with sEV^C^ from the same condition, Mann–Whitney *U* test. CM, conditioned medium; FI, fold induction

Next, we aimed to investigate the ex vivo effects of lipotoxic Hep-sEVs in mouse pancreatic islets. Incubation with sEV^PA^ promoted p65-NF-κB nuclear translocation in islet macrophages (positive for F4/80) 1 h after addition (Fig. 2a). Interestingly, nuclear p65-NF-κB immunostaining was visualised at 2 h in the cells surrounding the activated macrophages, beta cells among others. Also, treatment of islets with sEV^PA^ increased *Il1b* mRNA expression (Fig. 2b) and IL-1β levels in the culture medium (Fig. 2c). Next, islet macrophages were depleted using clodronate-loaded liposomes to determine their contribution to sEV^PA^-mediated islet inflammation. Figure 2d shows preserved morphology in clodronate-treated islets (CLOD islets). Of note, macrophage depletion in islets was assessed by F4/80 immunostaining and analysis of *Adgre1* transcript levels (Fig. 2d, e). Moreover, CLOD islets showed a marked downregulation of *Il1b* mRNA compared with control islets (Fig. 2f). Importantly, macrophage depletion in islets counteracted *Il1b* mRNA upregulation and IL-1β release to the medium induced by sEV^PA^ (Fig. 2b, c). These results suggest that macrophages are likely the dominant players in sEV^PA^-mediated proinflammatory responses in islets. Nevertheless, relative to *Il1b* mRNA in CLOD islets treated with sEV^C^, *Il1b* mRNA increased by eightfold upon treatment with sEV^PA^ (Fig. 2b), suggesting a macrophage-independent IL-1β release in islets upon sEV^PA^ stimulation. Furthermore, incubation with sEV^PA^ significantly lowered islet glucose responsiveness by reducing GSIS at high glucose concentration (16.7 mmol/l) (*p*<0.01) (Fig. 2g, h) without changes in insulin content (Fig. 2i). sEV^PA^ treatment also decreased islet *Pdx1*, *Ins1*, *Ins2* and *Gck* mRNA expression (Fig. 2j), reinforcing the deterioration of beta cell function. By contrast, macrophage depletion conferred protection against sEV^PA^-mediated decrease of GSIS (Fig. 2g, h) and *Pdx1*, *Ins1* and *Gck* mRNA downregulation (Fig. 2k).

### TLR4-dependent proinflammatory response in islet macrophages drives sEV^PA^-mediated alteration in insulin secretion in pancreatic islets

**Fig. 3:**
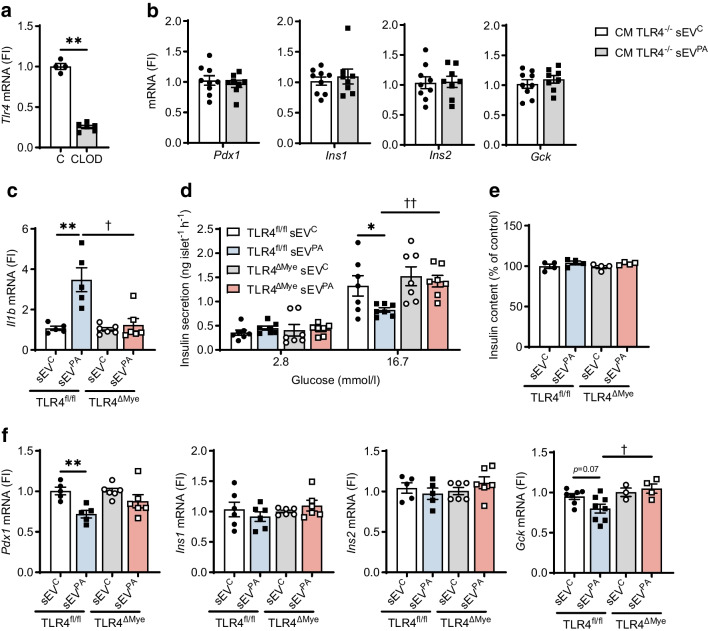
Effect of Hep-sEVs in TLR4^fl/fl^ and TLR4^∆Mye^ islets ex vivo. (**a**) *Tlr4* mRNA levels in control (C) and CLOD islets (C *n*=4, CLOD *n*=6). (**b**) Beta cell identity gene expression in INS-1 cells incubated with the CM from TLR4^−/−^ peritoneal macrophages treated with Hep-sEVs (sEV^C^ *n*=9, sEV^PA^ *n*=8) for 6 h. (**c**) *Il1b* mRNA levels in TLR4^fl/fl^ and TLR4^∆Mye^ islets treated with Hep-sEVs for 8 h (TLR4^fl/fl^ *n*=5, TLR4^∆Mye^ *n*=6). (**d**) GSIS (ng islet^−1^ h^−1^) by TLR4^fl/fl^ and TLR4^∆Mye^ islets treated with Hep-sEV for 24 h (*n*=7/group). (**e**) Insulin content (% of control) in TLR4^fl/fl^ and TLR4^∆Mye^ islets treated with Hep-sEVs for 24 h (*n*=4/group). (**f**) Beta cell identity gene expression in TLR4^fl/fl^ and TLR4^∆Mye^ islets treated with Hep-sEVs for 8 h (TLR4^fl/fl^ *n*=5–8, TLR4^∆Mye^ *n*=3–6). Data are expressed as the mean ± SEM. In (**a**): **p<0.01, compared with C, Mann–Whitney *U* test. In (**c** and **f**): **p<0.01, compared with sEV^C^ from the same genotype, Mann–Whitney *U* test; ^†^*p*<0.05, compared with sEV^PA^ in TLR4^fl/fl^ islets, Mann–Whitney *U* test. In (**d**): *p<0.05, compared with sEV^C^ at 16.7 mmol/l glucose from the same genotype; ^††^*p*<0.01, compared with sEV^PA^ in TLR4^fl/fl^ islets, two-way ANOVA and Bonferroni post hoc test. FI, fold induction

Recent studies pointed out TLR4-mediated inflammation in islet macrophages as a mechanism underlying beta cell dysfunction [11–13]. In this regard, our previous results demonstrated that sEV^PA^ act as SFAs carriers and trigger inflammation in peritoneal macrophages through the TLR4/NF-κB pathway [7]. Interestingly, as shown in ESM Fig. 4, sEV^PA^ are enriched in Fetuin-A, an adaptor ligand for SFA-mediated inflammation [14]. Of note, *Tlr4* expression was downregulated in CLOD islets (Fig. 3a). Therefore, macrophages from mice with global TLR4 deletion (TLR4^−/−^) were treated with sEV^PA^ for 24 h. The CM was then added to INS-1 cells for a further 6 h and gene expression analysis was performed. In contrast to the CM from wild-type macrophages treated with sEV^PA^ (Fig. 1g), CM from sEV^PA^-treated TLR4^−/−^ macrophages did not decrease *Pdx1* and *Gck* mRNAs in INS-1 cells (Fig. 3b).

To further determine the role of TLR4 signalling in macrophage-mediated islet inflammation by sEV^PA^, pancreatic islets from mice with specific TLR4 deletion in myeloid cells (TLR4^∆Mye^) and their respective controls (TLR4^fl/fl^) were isolated. As expected, incubation with sEV^PA^ elevated *Il1b* mRNA (Fig. 3c) and impaired GSIS (Fig. 3d) in TLR4^fl/fl^ islets without differences in insulin content (Fig. 3e). Likewise, sEV^PA^ treatment reduced *Pdx1* expression in TLR4^fl/fl^ islets (Fig. 3f). By contrast, islets from TLR4^∆Mye^ mice were protected against the elevation of *Il1b* expression (Fig. 3c), GSIS impairment (Fig. 3d) and *Pdx1* mRNA downregulation (Fig. 3f) by sEV^PA^. Also, islets from TLR4^∆Mye^ mice showed higher *Gck* mRNA levels upon treatment with sEV^PA^ compared with those from TLR4^fl/fl^ mice. No changes in *Ins1* and *Ins2* mRNA levels were found.

### Lipotoxic sEVs released by hepatocytes induce ER stress-mediated signalling and impair insulin secretion in beta cells

**Fig. 4:**
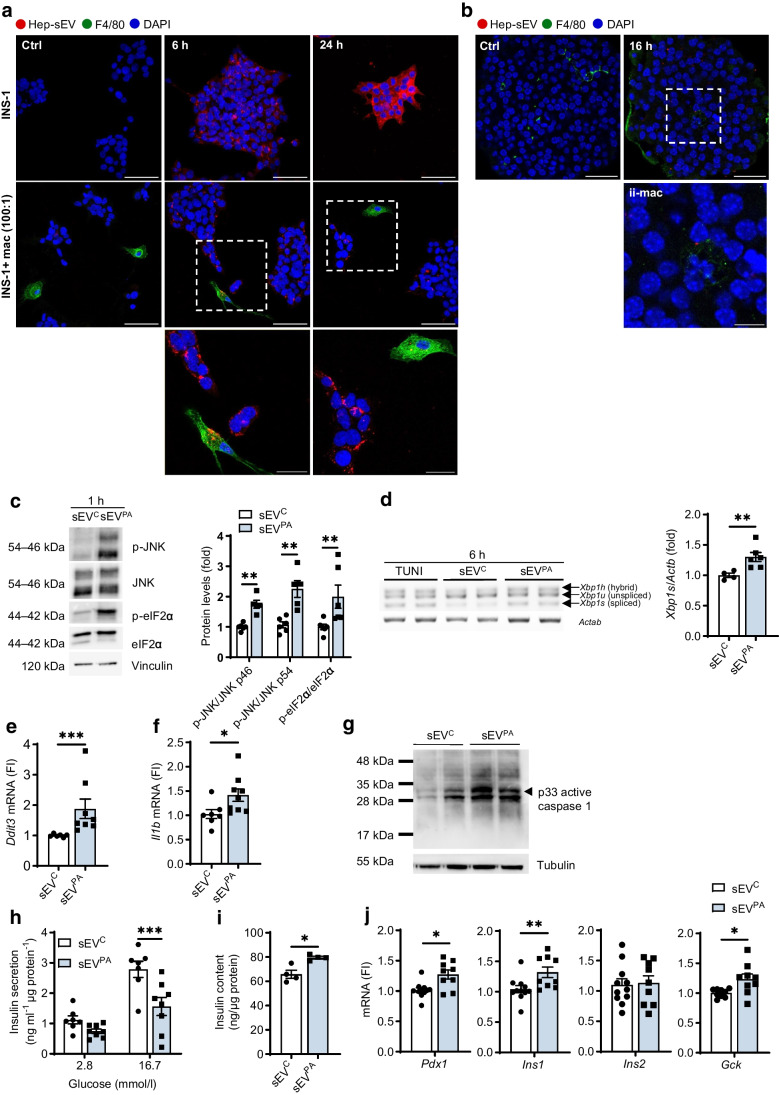
Effect of Hep-sEV treatment in INS-1 cells. (**a**) Representative images of PKH26-labelled sEV (red) uptake by INS-1 cells or INS-1 cells co-cultured with peritoneal macrophages (mac) (green) (100:1) for 6 and 24 h after addition and counterstained with DAPI (blue) (scale bars, 50 µm in upper and middle panels, and 20 µm in lower panels). (**b**) Representative images of PKH26-labelled sEV uptake ex vivo by mouse pancreatic islets (scale bars, 30 µm in upper panels and 10 µm in lower panel). (**c**) Representative western blot analysis with the indicated antibodies, p-JNK/JNK and p-eIF2α/eIF2α, and quantification, plotted as fold relative to sEV^c^ (*n*=5–6/group). (**d**) *Xbp1* splicing and quantification, plotted as fold relative to sEV^c^ (sEV^C^ *n*=4, sEV^PA^ *n*=6). Tunicamycin (TUNI) (10 µg/µl) was used as positive control. (**e**) *Ddit3* (sEV^C^ *n*=6, sEV^PA^ *n*=8) and (**f**) *Il1b* mRNA levels at 6 h after addition of sEVs (sEV^C^ *n*=7, sEV^PA^ *n*=9). (**g**) Representative western blot analysis of active caspase 1 (p33 fragment) of two independent experiments. (**h**) GSIS (ng ml^−1^ µg protein^−1^) (sEV^C^ *n*=7, sEV^PA^ *n*=8) and (**i**) insulin content (ng/µg protein) (*n*=4/group) at 24 h post addition of sEVs. (**j**) Beta cell identity gene expression (sEV^C^ *n*=9–11, sEV^PA^ *n*=9) at 6 h. Data are expressed as the mean ± SEM. In (**c**–**f**, **i** and **j**): *p<0.05, **p<0.01, ***p<0.001, compared with sEV^C^, Mann–Whitney *U* test. In (**h**): ***p<0.001, compared with sEV^C^ at 16.7 mmol/l glucose, two-way ANOVA and Bonferroni post hoc test. Ctrl, control; FI, fold induction

Since the results of Fig. 2b, c suggest a macrophage-independent IL-1β release induced by lipotoxic sEVs in pancreatic islets, we investigated potential direct effects of sEV^PA^ in beta cells. We first tested the ability of INS-1 beta cells to internalise Hep-sEVs when cultured alone or co-cultured with peritoneal macrophages at 100:1 ratio to mimic the estimated ratio of beta cells to macrophages in pancreatic islets [13]. Figure 4a shows the internalisation of Hep-sEVs by INS-1 cells at 6 h when they were cultured either alone or with macrophages. Fluorescence intensity was reduced in INS-1 cells co-cultured with macrophages indicating higher Hep-sEV internalisation capacity of the macrophages. Next, PKH26-labelled Hep-sEVs were incubated with pancreatic islets ex vivo for 16 h and fluorescence was found in both F4/80-positive and non-F4/80 cells (Fig. 4b). Incubation of INS-1 cells with sEV^PA^ did not alter cellular viability (ESM Fig. 1b), but triggered ER stress manifested by increases in JNK and eIF2α phosphorylation (Fig. 4c), *Xbp-1* splicing (Fig. 4d) and *Ddit3* expression (Fig. 4e) compared with INS-1 cells receiving sEV^C^. Neither JNK nor eIF2α phosphorylation was increased in INS-1 cells receiving sEV^C^ (ESM Fig. 2b). Notably, *Il1b* mRNA levels were also upregulated by sEV^PA^ (Fig. 4f). Likewise, incubation of INS-1 cells with sEV^PA^ increased caspase 1 p33 fragment (Fig. 4g), suggesting that *Il1b* upregulation is likely to occur through activation of the inflammasome. Direct addition of sEV^PA^ also reduced GSIS (Fig. 4h) and increased insulin content (Fig. 4i), as well as *Pdx1*, *Ins1* and *Gck* expression levels (Fig. 4j), without changes in PDX1 nuclear localisation (ESM Fig. 3b).

### Chronic administration of lipotoxic Hep-sEVs to lean mice increases circulating lipids and proinflammatory cytokines and induces hepatic inflammation and injury

**Fig. 5:**
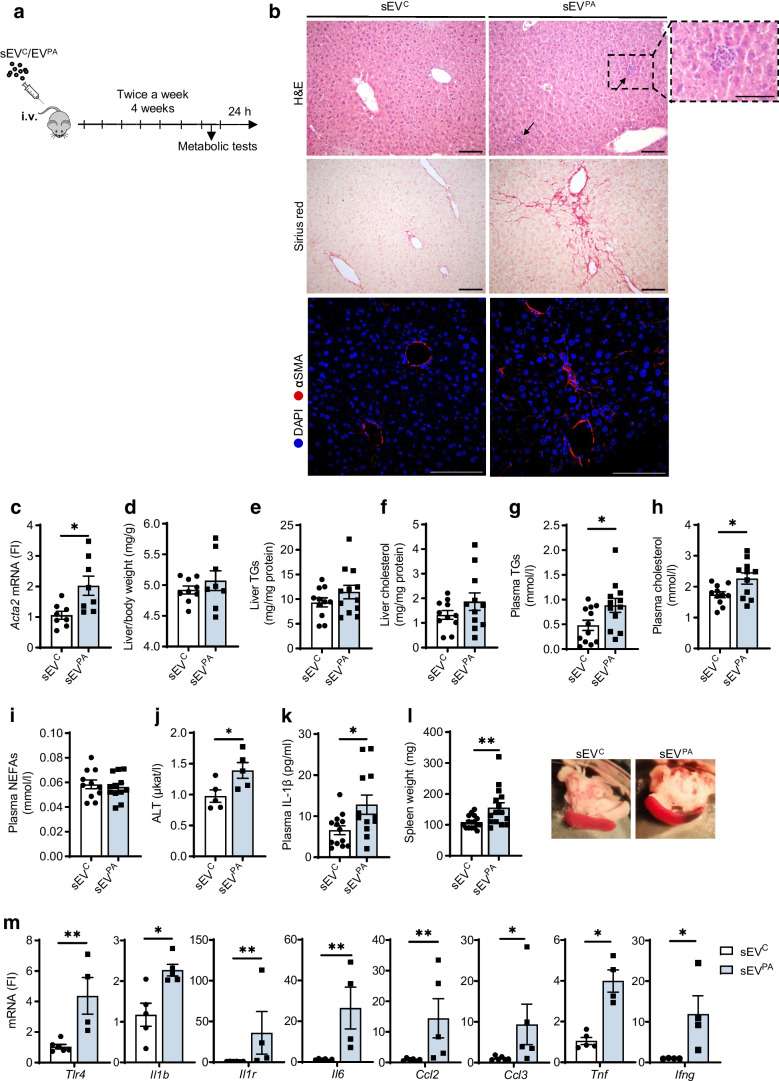
Effects of chronic Hep-sEV administration on liver and whole-body inflammation. (**a**) Experimental design of chronic Hep-sEV i.v. injections. (**b**) Representative images of H&E, Sirius red and immunofluorescence staining of αSMA (red) and counterstaining with DAPI (blue) of livers after chronic injections (scale bars, 100 µm; 50 µm in upper panel zoom). (**c**) *Acta2* mRNA levels in the liver (*n*=8/group). (**d**) Liver-to-body weight ratio (mg/g) (sEV^C^ *n*=9, sEV^PA^ *n*=8). (**e**) Liver TGs (sEV^C^ *n*=11, sEV^PA^ *n*=12) and (**f**) cholesterol (mg/mg protein) (*n*=11/group). (**g**) Plasma TGs (mmol/l) (*n*=12/group), (**h**) cholesterol (mmol/l) (*n*=11/group) and (**i**) NEFAs (mmol/l) (sEV^C^ *n*=11, sEV^PA^ *n*=12). (**j**) ALT activity (μkat/l) in plasma (*n*=5/group). (**k**) Plasma IL-1β levels (pg/ml) (sEV^C^ *n*=13, sEV^PA^ *n*=12). (**l**) Spleen weight (mg) (*n*=15/group) and representative gross pictures. (**m**) Immune-related gene expression in the liver (*n*=4–6/group). Data are expressed as the mean ± SEM. *p<0.05, **p<0.01, compared with sEV^C^, Mann–Whitney *U* test. FI, fold induction

To investigate the in vivo relevance of our findings we conducted a treatment with lipotoxic Hep-sEVs in mice. To achieve this, lean C57BL/6J male mice were intravenously injected with Hep-sEVs (sEV^C^ or sEV^PA^) biweekly for 4 weeks. Mice were culled 24 h after the last injection (Fig. 5a) and the liver and pancreas were analysed.

Inflammatory foci and fibrillar collagen deposition were observed in livers from sEV^PA^-injected mice (Fig. 5b), as well as an upregulation of *Acta2* mRNA (Fig. 5c) and α smooth muscle actin (αSMA) protein immunostaining (Fig. 5b), these features being characteristic of MASH. No differences between groups were found in liver-to-body weight ratio or hepatic TG or cholesterol levels (Fig. 5d–f). Treatment with sEV^PA^ elevated circulating TG and total cholesterol without changes in plasma NEFAs (Fig. 5g–i). Notably, plasma ALT was increased by sEV^PA^ injections, suggesting hepatic parenchymal damage (Fig. 5j). In addition, increased plasma levels of the proinflammatory cytokine IL-1β were found in mice treated with sEV^PA^ (Fig. 5k). In this line, sEV^PA^-treated mice presented with enlarged spleen compared with mice injected with sEV^C^ (Fig. 5l), an effect probably due to liver injury and systemic inflammation. Likewise, elevated immune-related gene expression (*Tlr4*, *Il1b*, *Il1r*, *Il6*, *Ccl2*, *Ccl3*, *Tnf* and *Ifng*) was found in liver from mice injected with sEV^PA^ (Fig. 5m).

**Fig. 6:**
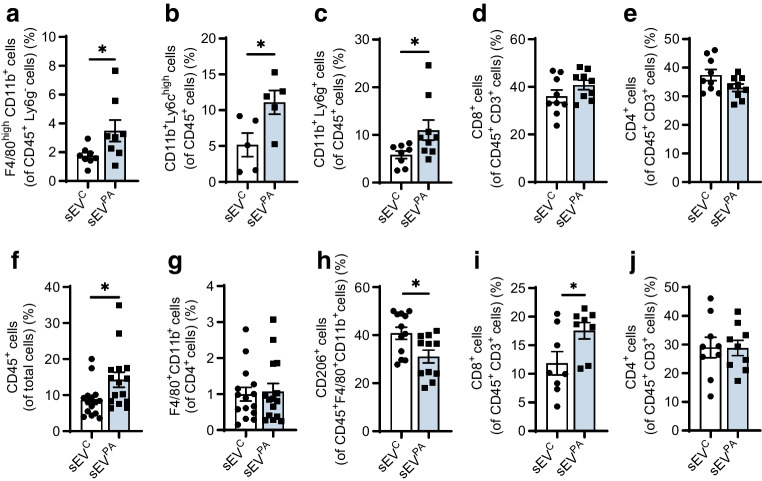
Effects of chronic Hep-sEV administration on hepatic and pancreatic immune cells. (**a**–**e**) Hepatic immune cells. (**a**) Percentages of Kupffer cells (CD45^+^ Ly6g^−^ F4/80^high^ CD11b^+^ cells) (*n*=8/group), (**b**) monocytes (CD45^+^ CD11b^+^ Ly6c^high^ cells) (*n*=5/group), (**c**) neutrophils (CD45^+^ CD11b^+^ Ly6g^+^ cells) (sEV^C^ *n*=8, sEV^PA^ *n*=9), (**d**) cytotoxic T lymphocytes (CD45^+^ CD3^+^ CD8^+^ cells) (*n*=9/group) and (**e**) T helper lymphocytes (CD45^+^ CD3^+^ CD4^+^ cells) (*n*=9/group). (**f**–**j**) Pancreatic immune cells. (**f**) Percentages of leucocytes (CD45^+^ cells) (*n*=15/group), (**g**) total macrophages (CD45^+^ F4/80^+^ CD11b^+^ cells) (*n*=15/group), (**h**) M2 macrophages (CD45^+^ F4/80^+^ CD11b^+^ CD206^+^ cells) (sEV^C^ *n*=12, sEV^PA^ *n*=11), (**i**) T cytotoxic lymphocytes (CD45^+^ CD3^+^ CD8^+^ cells) (sEV^C^ *n*=8, sEV^PA^ *n*=8) and (**j**) T helper lymphocytes (CD45^+^ CD3^+^ CD4^+^ cells) (sEV^C^ *n*=9, sEV^PA^ *n*=9). Data are expressed as the mean±SEM. *p<0.05, compared with sEV^C^, Mann–Whitney *U* test

To investigate changes in the hepatic immune environment during chronic Hep-sEV administration, we isolated liver NPCs and immunophenotyping was performed by flow cytometry (ESM Fig. 5a). Figure 6a shows a higher percentage of F4/80^high^ CD11b^+^ cells (Kupffer cells) in sEV^PA^-treated mice compared with those receiving sEV^C^. Furthermore, a significant increase in the percentages of CD11b^+^ Ly6c^+^ cells (monocytes) and CD11b^+^ Ly6g^+^ cells (neutrophils) was observed in mice treated with sEV^PA^ (Fig. 6b, c). Regarding lymphocyte populations, no differences were found in CD8^+^ (T cytotoxic) and CD4^+^ (T helper) cells (Fig. 6d, e). In agreement, liver immunofluorescence showed infiltration of macrophages organised in crown-like structures across the tissue, as well as monocytes and neutrophils in sEV^PA^-injected mice (ESM Fig. 6).

### sEVs released from lipotoxic hepatocytes enhanced pancreatic inflammation

We further analysed the profile of infiltrating immune cells in the pancreas of mice receiving chronic Hep-sEV injections (ESM Fig. 5b). As shown in Fig. 6f, sEV^PA^-treated mice exhibited an increase in the percentage of total pancreatic immune (CD45^+^) cells. Moreover, while the total macrophage pool (F4/80^+^ CD11b^+^ cells) did not differ between groups (Fig. 6g), M2-like polarised macrophages (CD206^+^ cells) were reduced in mice receiving sEV^PA^ (Fig. 6h). In addition, recruitment of CD8^+^ T cells was found in the pancreas from sEV^PA^-injected mice (Fig. 6i) without changes in the CD4^+^ T subpopulation (Fig. 6j).

### Effect of the chronic treatment of lean mice with lipotoxic Hep-sEVs on glucose homeostasis

**Fig. 7:**
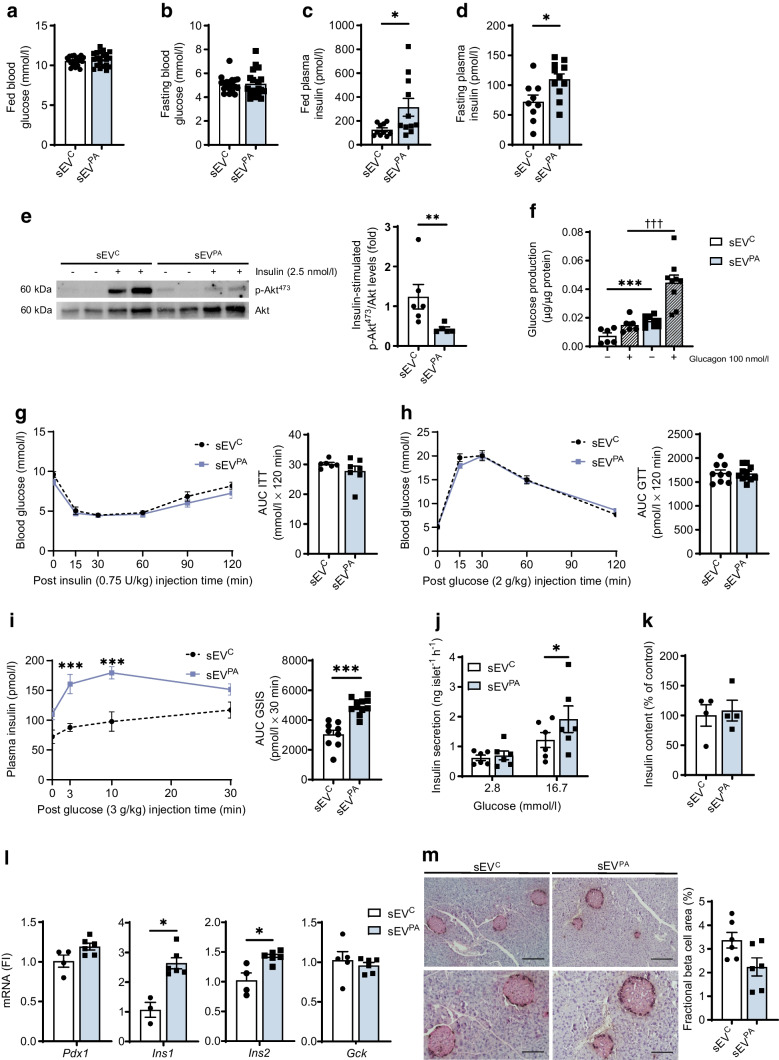
Effects of chronic Hep-sEV administration on glucose homeostasis and insulin action and secretion. (**a**) Fed (sEV^C^ *n*=20, sEV^PA^ *n*=21) and (**b**) fasting blood glucose (mmol/l) (*n*=19/group). (**c**) Fed and (**d**) fasting plasma insulin (pmol/l) (sEV^C^ *n*=9–10, sEV^PA^ *n*=11). (**e**) Akt phosphorylation levels (Ser473) normalised to total Akt protein in primary hepatocytes. Representative western blots and quantification (plotted as fold vs sEV^C^) are shown (sEV^C^ *n*=6, sEV^PA^ *n*=5). (**f**) Glucose production in primary hepatocytes under basal condition and after glucagon stimulation (sEV^C^ *n*=6, sEV^PA^ *n*=9). (**g**) ITT (0.75 U/kg i.p. injection) and respective AUC (sEV^C^ *n*=6, sEV^PA^ *n*=7). (**h**) GTT (2 g glucose/kg i.p. injection) and respective AUC (sEV^C^ *n*=9, sEV^PA^ *n*=11). (**i**) In vivo GSIS (3 g glucose/kg i.p. injection) and respective AUC (sEV^C^ *n*=9, sEV^PA^ *n*=11). AUC was calculated from 0 to 120 min (for ITT and GTT) and from 0 to 30 min (for GSIS) values according to the trapezoidal rule. (**j**) Ex vivo GSIS by isolated pancreatic islets (ng islet^−1^ h^−1^). Experiments were performed with eight technical replicates per mouse (sEV^C^ *n*=6, sEV^PA^ *n*=6). (**k**) Insulin content (% of control) in pancreatic islets (*n*=4/group). (**l**) Beta cell identity gene expression in pancreatic islets (sEV^C^ *n*=3–5, sEV^PA^ *n*=6). (**m**) Representative images of islets stained with insulin and glucagon (scale bars, 200 µm [upper panel] or 100 µm [lower panel]), and fractional beta cell area quantification (*n*=6/group). The percentage of beta cell area was analysed and expressed relative to the total pancreatic area measured in the sections of each condition. Lower panels show an area of the upper panels. Data are expressed as the mean ± SEM. In (**c**, **d**, **e**, **i** [AUC graph] and **l**): *p<0.05, **p<0.01, ***p<0.001, compared with sEV^C^, Mann–Whitney *U* test. In (**f**): ***p<0.001, compared with sEV^C^ in basal conditions; ^†††^*p*<0.001, compared with sEV^C^ after glucagon stimulation, Mann–Whitney *U* test. In (**i**) (left graph): ***p<0.001, compared with sEV^C^, one-way ANOVA and Bonferroni Post hoc test. In (**j**): *p<0.05, compared with sEV^C^ at 16.7 mmol/l glucose, two-way ANOVA and Bonferroni post hoc test. FI, fold induction

Since chronic treatment with lipotoxic Hep-sEVs induced liver and pancreas inflammation in mice, we evaluated glucose homeostasis. As shown in Fig. 7a, b, there were no changes in fed or fasting blood glucose levels in sEV^PA^-treated mice. However, those mice presented higher plasma insulin in both fed and fasting states (Fig. 7c, d). Consistently, the analysis of insulin signalling revealed decreased insulin-induced AKT phosphorylation in primary hepatocytes isolated from sEV^PA^-injected mice at the end of the treatment in comparison with hepatocytes from mice receiving sEV^C^ (Fig. 7e). Moreover, higher glucose production under basal conditions and in response to glucagon was found in primary hepatocytes from sEV^PA^-injected mice (Fig. 7f). However, there were no differences between groups either in the ITT (Fig. 7g) or in the GTT (Fig. 7h). Surprisingly, in vivo GSIS increased in sEV^PA^-treated mice (AUC ***p<0.001) (Fig. 7i) compared with mice receiving sEV^C^. Consistently, static incubation of pancreatic islets isolated from sEV^PA^-treated mice revealed a twofold increase in insulin secretion when challenged with high glucose (16.7 mmol/l) compared with islets from mice receiving sEV^C^ (Fig. 7j) without changes in islet insulin content (Fig. 7k). Moreover, *Ins1* and *Ins2* mRNA levels were upregulated in islets from sEV^PA^-injected mice (Fig. 7l). Notably, no alterations in beta cell fractional area, apoptosis and cell proliferation were found (Fig. 7m, ESM Fig. 7). Altogether, these results suggest that sEV^PA^-treated mice develop insulin resistance in hepatocytes without alterations in glucose homeostasis, pointing to a compensatory insulin secretion by the beta cells in vivo.

### Lipotoxic sEVs from Huh7 human hepatocytes impaired insulin secretion in human islets

**Fig. 8:**
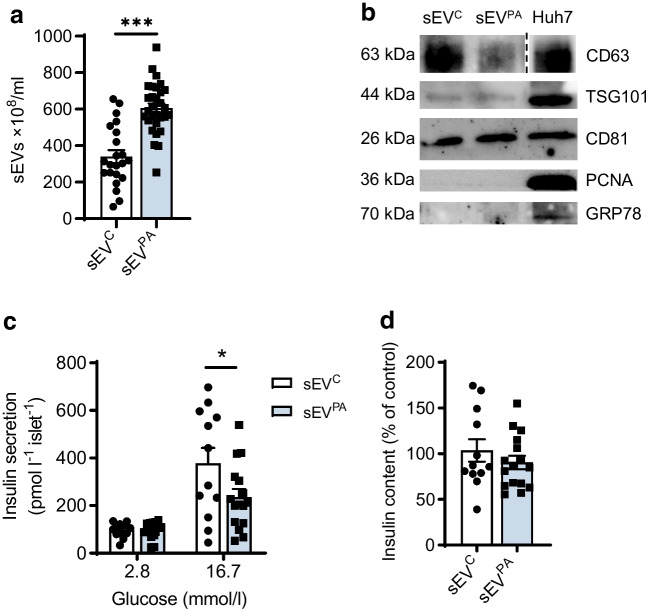
Effects of human Hep-sEVs on human pancreatic islet insulin secretion. (**a**) Concentration of sEVs secreted by Huh7 human hepatocytes after stimulation with 200 µmol/l PA (sEV^PA^) or BSA (sEV^C^) for 24 h evaluated by NTA (sEV^C^ *n*=22, sEV^PA^ *n*=30 from four independent Hep-sEV isolations). (**b**) Specific markers (CD63, TSG101, CD81) and negative markers (PCNA, GRP78) of sEVs analysed by western blot. sEVs from three independent isolations were pooled and analysed. Huh7 cells were used as controls for sEV negative markers. (**c**) GSIS (pmol l^−1^ islet^−1^) by human pancreatic islets treated for 24 h with sEVs released by Huh7 human hepatocytes (sEV^C^ *n*=12, sEV^PA^ *n*=16). (**d**) Insulin content (% of control) in human islets treated with sEVs for 24 h (sEV^C^ *n*=12, sEV^PA^ *n*=16). Data are expressed as the mean ± SEM. In (**a**): ***p<0.001, compared with sEV^C^, Mann–Whitney *U* test. In (**c**): *p<0.05, compared with sEV^C^ at 16.7 mmol/l glucose, two-way ANOVA and Bonferroni post hoc test

To add translational value to our results we isolated sEVs from Huh7 human hepatocytes treated with PA (200 µmol/l) or BSA for 24 h. NTA revealed that lipotoxic Huh7 human hepatocytes secreted a higher number of sEVs compared with the control condition (Fig. 8a). sEV-specific markers (CD63, CD81, TSG101) were also characterised by western blot (Fig. 8b). As observed in mice, sEV^PA^ released by lipotoxic human hepatocytes decreased GSIS in human islets (*p*<0.05) (Fig. 8c) without changes in insulin content (Fig. 8d).

## Discussion

Previous studies in the context of insulin resistance linked to obesity have been focused on the impact of adipose tissue-derived sEVs in the pancreas [15, 16]. Herein, ex vivo experimental approaches demonstrated that islet macrophages, via TLR4-dependent inflammation, mediate the deleterious effects of lipotoxic Hep-sEVs on beta cell function. Furthermore, we analysed the systemic effects in mice following chronic administration of these lipotoxic sEVs to elucidate their role in the pathogenesis of type 2 diabetes.

Macrophages are the dominant immune cells driving islet inflammation in type 2 diabetes [17]. Importantly, in addition to reaching the liver [7], in the present study we confirmed that Hep-sEVs also target the pancreas and accumulate in the macrophages in both endocrine (intra- and peri-islet) and exocrine areas 24 h after i.v. injection. Although a recent study already detected sEV enrichment in the pancreas after i.v. injection by intravital microscopy [15], this is the first study identifying macrophages as targets of Hep-sEVs in this organ. Only two previous reports have assessed the effects of Hep-EVs from obese mice on beta cell proliferation [5] and apoptosis [6]. Herein, we demonstrate that, in isolated pancreatic islets, sEV^PA^ increased *Il1b* expression and IL-1β release, a cytokine relevant in islet inflammation [18]. Importantly, sEV^PA^ decreased islet GSIS, an effect partly attributed to a reduction in beta cell identity gene expression. Immunostaining showed p65-NF-κB nuclear translocation in islet macrophages followed by its presence in cells surrounding the activated macrophages, mainly beta cells, indicating that the inflammatory response in the islets is likely driven by the macrophages and propagates to the nearby beta cells. In this context, the CM released by macrophages exposed to lipotoxic Hep-sEVs, containing IL-1β as we previously reported [7], diminished GSIS as well as *Pdx1* and *Gck* expression in INS-1 beta cells. In addition, depletion of islet macrophages with clodronate-loaded liposomes counteracted sEV^PA^-induced *Il1b* upregulation, GSIS decline and downregulation of beta cell identity genes. Previous studies using this approach have also shown GSIS recovery in islets from a mouse model of diabetes [13] and obese mice [19]. Taken together, our data suggest that lipotoxic Hep-sEVs impair ex vivo GSIS by activating macrophage-dependent islet inflammation. The deleterious impact of lipotoxic Hep-sEVs on ex vivo GSIS in mouse pancreatic islets was also found in human pancreatic islets exposed to sEVs released by lipotoxic human hepatocytes, highlighting the translational value of our results.

SFA-mediated islet inflammation impairs beta cell function via TLR4 signalling [13] and, consistently, TLR4 deficiency protects pancreatic islets against the deleterious effects of obesity [20–23]. Other studies show that macrophages are a dominant source of TLR4 expression in islets from healthy mice and humans [11], as well as in islets from individuals with diabetes [12]. Herein, we found that in pancreatic islets from TLR4^∆Mye^ mice the deleterious effects of sEV^PA^ on* Il1b* upregulation, beta cell identity gene downregulation and GSIS impairment were attenuated, demonstrating the relevance of macrophage TLR4 in lipotoxic Hep-sEV effects in pancreatic islets. Considering that PA and Fetuin-A stimulate TLR4-dependent IL-1β secretion in islet macrophages [24] and that Hep-sEVs are enriched in both PA [7] and Fetuin-A (this study) under MASLD conditions, our results suggest that sEVs may act as shuttles of SFA or/and Fetuin-A and induce TLR4-dependent IL-1β secretion from both liver and islet macrophages, thereby promoting beta cell dysfunction and type 2 diabetes development.

Besides the interaction with islet macrophages, our results show that beta cells are able to internalise Hep-sEVs which, in turn, activates the proapoptotic unfolded protein response via protein kinase R-like ER kinase (PERK)/eIF2α, inositol-requiring enzyme 1 (IRE1)/X-box binding protein 1 (XBP-1) and IRE1/JNK and the inflammasome, resulting in GSIS impairment. Although chronic ER stress in beta cells reduces insulin transcription and translation [25], our results showed increased *Pdx1*, *Ins1* and *Gck* expression and insulin content in INS-1 cells following sEV^PA^ treatment. Nonetheless, depletion of ER Ca^2+^ is another consequence of prolonged ER stress in beta cells [25], suggesting that the effect of lipotoxic Hep-sEVs on GSIS likely occurs by disrupting the insulin secretory machinery, an issue that deserves further investigation. In addition, several studies demonstrated that ER stress can activate the NLR family pyrin domain containing 3 (NLRP3) inflammasome and induce IL-1β production in beta cells [26]. Therefore, it seems likely that in beta cells lipotoxic Hep-sEVs trigger IL-1β secretion via ER stress/NLRP3 inflammasome. Thus, a direct effect of lipotoxic sEVs in beta cell dysfunction cannot be excluded since they express TLR4, as reported [27], and TLR4 is upregulated in beta cells from obese mice [28]. This possibility is supported by our results showing elevated *Il1b* expression in pancreatic islets depleted of macrophages exposed to sEV^PA^ compared with those receiving sEV^C^. However, we cannot conclude whether IL-1β was secreted by the beta cells and/or other islet cells (e.g. alpha cells) [29].

This study also provides in vivo findings showing liver and pancreas inflammation in lean mice receiving sEV^PA^ for 4 weeks that developed a MASH-like inflammatory phenotype without steatosis. Regarding the liver, inflammation induced by lipotoxic sEVs was characterised by monocyte and neutrophil recruitment and by an increase in the number of macrophages organised in crown-like structures. All these features are associated with MASH in mice and humans [30, 31]. Furthermore, this intervention induced fibrotic damage in line with previous in vitro studies, supporting the role of lipotoxic Hep-sEVs in promoting activation of hepatic stellate cells during MASH [32], as well as hepatocyte injury. Of relevance, in parallel with hepatic MASH-like features, mice receiving sEV^PA^ showed an increase in infiltrating immune cells in the pancreas, particularly CD8^+^ T lymphocytes. In agreement, a rise in CD8^+^ T cells was found in individuals with type 2 diabetes by single-cell analysis in both intra- and peri-islet areas and in the exocrine pancreas [33]. Since macrophages and macrophage-derived proinflammatory cytokines and chemokines are increased in human and mouse islets in type 2 diabetes [13, 19, 33–37], our results suggest that M1-like macrophages could drive the recruitment of CD8^+^ T cells in response to sEV^PA^. However, despite the reduction in M2-like macrophages in the pancreas of mice receiving those lipotoxic sEVs, likely due to M1-like polarisation, both groups of mice showed similar total number of macrophages. Of relevance, in type 2 diabetes, enrichment in M1-like macrophages is restricted to intra-islet areas [33] which represent only 1–4% of the total pancreatic volume [38]. Therefore, further analysis discriminating macrophages by pancreatic area is needed to elucidate whether sEV^PA^ increase their number in the intra-islet location.

In addition to the local effects in the liver and pancreas, we found that lipotoxic Hep-sEVs also induced systemic effects including increases in fed and fasting insulin, plasma TG, cholesterol and IL-1β, all risk factors associated with dysregulated glucose homeostasis and insulin secretion, as well as insulin resistance [39]. Although no differences in whole-body glucose tolerance were observed between groups, mice chronically treated with sEV^PA^ showed enhanced GSIS both in vivo and in isolated islets accompanied by increased expression of islet identity genes. These findings are in line with longitudinal studies reporting an initial compensatory period of enhanced insulin secretion without changes in glucose levels in insulin-resistant individuals [40], particularly in individuals with prediabetes and MASLD [41]. Consistent with the increase in plasma insulin levels, chronic treatment with sEV^PA^ attenuated insulin signalling and increased glucose production in hepatocytes. Altogether, these results point to an early state of hepatocyte insulin resistance that leads to a compensatory beta cell-mediated insulin secretion in mice receiving sEV^PA^. This compensatory response occurs in the absence of islet hyperplasia or alterations in islet architecture. Nonetheless, we cannot exclude other mechanisms by which lipotoxic Hep-sEVs might affect islet functionality in vivo such as beta cell insulin resistance. In this regard, a recent study in mice with beta cell-specific insulin receptor (*Insr*) deletion under the *Ins1* promoter, currently the most beta cell-specific Cre deletion strain available, showed insulin hypersecretion and improved glucose tolerance, even preceding the onset of global insulin resistance [42]. Notably, systemic insulin resistance and glucose intolerance have been found in lean mice receiving circulating sEVs or in adipose tissue-derived sEVs from obese mice and humans [43–47]. On the other hand, Ji et al [48] reported that administration of Hep-sEVs from obese mice to lean mice for a longer period of time decreased glucose tolerance and insulin sensitivity, although no parameters related to beta cell function were analysed in this previous study. Considering these data, extending the treatment with sEV^PA^ in our experimental settings may lead to glucose intolerance and/or more profound peripheral and beta cell insulin resistance. Another limitation of our study is the lack of in vivo data in TLR4^∆Mye^ mice chronically treated with lipotoxic Hep-sEVs, which would provide valuable mechanistic information.

It is noteworthy to point out that in vivo data regarding GSIS might seem contradictory to results in pancreatic islets ex vivo. While chronic administration of lipotoxic Hep-sEVs in mice induced beta cell compensatory increase in GSIS, incubation of pancreatic islets or INS-1 beta cells with sEV^PA^ led to beta cell failure. Besides differences between in vitro and in vivo experimental conditions such as sEV concentration and/or availability, a plausible explanation for these different responses might rely on endocrine components contributing to the beta cell compensatory response in mice receiving lipotoxic Hep-sEVs that are absent in the ex vivo settings. Growing evidence suggests that liver-derived circulating factors secreted in insulin-resistant states, including hepatocyte growth factor [49] or leukocyte-neutrophil elastase inhibitor [50], contribute to this effect. Also, in response to beta cell failure, macrophages secrete cytokines [51] and growth factors such as insulin-like growth factor 1 [52] that increase beta cell proliferation which raises the possibility that, in the in vivo context, islet macrophages might display a protective mechanism upon chronic lipotoxic Hep-sEV administration. Hence, apart from the effects on hepatocyte insulin resistance and liver inflammation, lipotoxic sEVs may also have an effect on the liver secretome. This hypothesis is supported by work from Kulkarni's laboratory in LIRKO mice showing liver-derived factors that promote the expansion of beta cell mass [53].

In summary, our results have provided evidence of the effect of lipotoxic Hep-sEVs in inducing liver and pancreas inflammation and beta cell dysfunction. Moreover, our data envision TLR4-mediated signalling in islet macrophages as a key mediator of the effects of lipotoxic Hep-sEVs on beta cell function. Thus, TLR4 could represent an attractive therapeutic target not only for the early treatment of MASLD, but also for preventing type 2 diabetes development by targeting islet macrophages.

## Supplementary Information

Below is the link to the electronic supplementary material.

ESM (PDF 1144 KB)

## Data Availability

Data presented in this manuscript are available upon request from the corresponding authors.

## Abbreviations

ALT: Alanine aminotransferase
CLOD islets: Clodronate-treated islets
CM: Conditioned medium
CM-sEV^C^: CM collected from macrophages treated with sEV^C^
CM-sEV^PA^: CM collected from macrophages treated with sEV^PA^
eIF2α: Eukaryotic translation initiation factor 2α
ER: Endoplasmic reticulum
EV: Extracellular vesicle
GRP78: Glucose regulated protein 78
GSIS: Glucose-stimulated insulin secretion
HBSS: Hanks' Balanced Salt Solution
Hep-sEV: Hepatocyte-derived sEV
ii-mac: Intra-islet macrophage
IKBα: NF-κB inhibitor α
JNK: c-Jun N-terminal kinase
MASH: Metabolic dysfunction-associated steatohepatitis
MASLD: Metabolic dysfunction-associated steatotic liver disease
NLRP3: NLR family pyrin domain containing 3
NPC: Non-parenchymal cell
NTA: Nanoparticle tracking analysis
PA: Palmitic acid
PCNA: Proliferating cell nuclear antigen
PDX1: Pancreatic and duodenal homeobox 1
PFA: Paraformaldehyde
pi-mac: Peri-islet macrophage
RT-qPCR: Reverse transcription real-time quantitative PCR
sEV: Small extracellular vesicle
sEV^C^: sEVs isolated from hepatocytes in the absence of PA
sEV^PA^: sEVs isolated from hepatocytes in the presence of PA
SFA: Saturated fatty acid
αSMA: Alpha smooth muscle actin
TG: Triglyceride
TLR4: Toll-like receptor 4
TSG101: Tumour susceptibility gene 101
