# Supplementary material for: Effect of lipotoxic hepatocyte-derived extracellular vesicles in pancreas inflammation: essential role of macrophage TLR4 in beta cell functionality
Source: Diabetologia. 2025 May 19;68(8):1801–22. doi: 10.1007/s00125-025-06445-z (PMC12245964; doi:10.1007/s00125-025-06445-z)
Supplement: Supplementary file 1 — ESM (PDF 1144 KB) [file 125_2025_6445_MOESM1_ESM.pdf]

ELECTRONIC SUPPLEMENTARY MATERIAL (ESM)

ESM METHODS

Isolation and culture of primary hepatocytes. After anaesthesia, the suprahepatic vena cava was cannulated through the atrium of the heart and mouse liver was perfused with two solutions pre-warmed in a water bath at 37°C. First, washing solution containing EGTA was perfused (25 ml, pump speed 3 ml/min) to wash out blood and circulating cells from the liver as well as to eliminate calcium. Then, collagenase solution was perfused (25 ml, pump speed 4 ml/min) to the liver to dissociate extracellular matrix, thereby facilitating cell dispersion. The liver was dissected and transfer to a petri dish with hepatocyte attachment medium (AM) containing DMEM and Ham's F-12 medium (1:1) with heat-inactivated 10% FBS, supplemented with 2 mM glutamine, 15 mM glucose, 20 mM HEPES, 100 U/ml penicillin, 100 µg/ml streptomycin and 1 mM sodium pyruvate, and cells were gently released using 1 ml tips. The suspension was filtered through a 100 µm-cell strainer into 50 ml-tube to proceed to purification of the hepatocytes by density-based separation. Cells were centrifuged at 50 × g for 5 min at 4 °C. The pellet containing hepatocytes was re-suspended by swirling the tube with AM and Percoll solution (GE17-0891-01, Sigma-Aldrich, San Louis, MA, USA) 1:1. The suspension was mixed thoroughly by inverting the tube several times and centrifuged again at 200 × g for 10 min at room temperature. Then, the pellet containing live purified hepatocytes was re-suspended in AM by swirling the tube and washed for the last time at 50 × g for 5 min at 4°C. Finally, hepatocytes were seeded on collagen (C3867-1VL, Sigma-Aldrich, San Louis, M, USA) pre-coated plates and maintained in AM for 24 h before treatments.

Isolation, culture and generation of conditioned medium from peritoneal macrophages. Peritoneal macrophages from thioglycolate-elicited mice were isolated using a quick peritoneal lavage method. Following euthanasia, the outer skin of the peritoneum was cut and gently pulled back to expose the inner skin lining the peritoneal cavity. Then, PBS was injected in the peritoneal cavity and the abdominal walls massaged to dislodge any attached peritoneal cells. PBS was draw back from the peritoneal cavity and collected. This is the peritoneal lavage that contains the peritoneal macrophages and other peritoneal cells. Cells were spined 500 × g for 5 min at 4 ºC, then, counted and cultured in RPMI medium supplemented with 10% heat-inactivated FBS, 100 U/ml penicillin, 100 µg/ml streptomycin. One hour after plating, macrophages were washed with PBS and incubated in RMPI medium containing 2% heat-inactivated FBS, 100 U/ml penicillin, 100 µg/ml streptomycin for 16 h before treatment. Experiments were performed in RPMI medium supplemented with 2% EV-depleted FBS, which was generated by ultracentrifugation of FBS for 18 h at 100,000 × g and 4 ºC using a 60Ti fixed-angle rotor.

Peritoneal macrophages from C57BL/6J or from TLR4$^{-/-}$ male mice were stimulated for 24 h with Hep-sEV and conditioned medium (CM) were collected and immediately frozen at -80°C for further stimulation of INS-1 beta cells. A total of 300 microliters of macrophage-derived CM were added to INS-1 cells seeded in a 12 well plate.

Insulin signalling and glucose production by primary hepatocytes. Primary hepatocytes isolated from mice after chronic treatment with sEV were maintained in culture for 24 h. After 1 h of starvation in serum-free DMEM medium with 5.5 mM glucose, hepatocytes were treated with 2.5 nM insulin (I0516, Sigma-Aldrich, USA) for 10 min. Then cells were washed with PBS, lysed and total protein lysates were analysed by Western blot.

For the analysis of glucose production, primary hepatocytes were incubated in serum-free, phenol-free DMEM medium with 5.5 mM glucose and 1mM glutamine for 3 h. Then, cells were washed twice to remove glucose from the culture plates and further stimulated with glucagon (100 nM) in glucose production medium (DMEM without glucose and phenol red, 1 mM glutamine, 2 mM sodium pyruvate, 20 mM sodium lactate) for 16 h. The glucose production in the culture medium was determined by the glucose oxidase-peroxidase method (Biosystems, Spain) according with the manufacturer's instructions. Glucose levels were referred to total protein content of each culture plate.

Immunostaining procedures

Fluorescent immunocytochemistry in INS-1 cells. Cells seeded in coverslips were fixed with 4% paraformaldehyde (PFA), permeabilized 2 min with 0.1% Triton X-100 (T8787, Sigma-Aldrich, USA) in PBS, blocked with 3% BSA in 0.1% Triton X-100 in PBS for 1h at RT and primary antibodies were applied in blocking buffer overnight at 4 ºC. For nuclei staining, cells were permeabilized with 0.3% Triton X-100 in PBS for 3 min. Then, cells were incubated for 1 h with the secondary antibodies conjugated to Alexa Fluor (Molecular Probes, Eugene, OR, USA), counterstained with DAPI and mounted with Fluoromount-G Mounting Medium (00-4958-02, Thermo Fisher Scientific, Waltham, MA, USA). Images were acquired with an LSM710 confocal microscope (Zeiss, Oberkochen, Germany) and quantified using ImageJ 2.0 Software (NIH). Cells with nuclear translocation of p65-NFκB were counted and expressed as a percentage of the total cell number.

In toto immunofluorescence of pancreatic islets. Hep-EV-treated pancreatic islets were handpicked and placed into micro-slide chambers (80826, Ibidi, Gräfelfing, Germany) containing PBS, washed 3 times with PBS by pipetting and fixed with 4% PFA for 5 min at RT. Islets were washed 3 times with PBS and permeabilized 30 min with 1% Triton X-100 in PBS. Islets were washed again 3 times with PBS and blocked with 3% BSA, 3% normal donkey serum in PBS for 1 h at RT. Primary antibodies

were applied in blocking buffer overnight at 4 ºC. After washing another 3 times with PBS, islets were incubated in slow agitation for 2 h at RT with Alexa Fluor conjugated secondary antibodies and counterstained with DAPI. Islets were washed for the last time 3 times with PBS and mounted with Fluoromount-G Mounting Medium (00-4958-02, Invitrogen, Waltham, MA, USA). Images were acquired with an LSM710 confocal microscope (Carl Zeiss, Oberkochen, Germany).

Fluorescent immunohistochemistry in liver and pancreatic tissue. Livers and pancreas were dissected and fixed in 4% PFA overnight at 4ºC. Livers and pancreas were transferred to 15% sucrose and 30% sucrose in PBS at 4 ºC until tissue sunk and then embedded in Tissue-tek OCT (2024-03, Sakura Finetek, Torrance, CA, USA). Cryosections of 5 or 6 µm thickness were cut for liver or pancreas, respectively. Cryostat sections were fixed in 4% PFA 20 min at -20 ºC upon removal from -80 ºC, then blocked with 6% BSA, 2% horse serum in PBS for 1h at RT. Primary antibodies were applied in 6% BSA, 2% horse serum, 0.3% Triton X-100 in PBS o/n at 4 ºC, then with their respective Alexa Fluor-conjugated secondary antibodies at RT for 1 h, and counterstained with DAPI. Random fields at 40x magnification (6 per mice) for αSMA were evaluated using an LSM710 confocal microscope (Carl Zeiss, Oberkochen, Germany) and quantified using ImageJ 2.0 Software (NIH). Random fields at 20x for F4/80, Clec4f, Ly6c and S100A9 were evaluated using Eclipse 90i microscope (Nikon Eclipse TS100) and quantified using ImageJ 2.0 Software (NIH). For anti-F4/80, anti-Clec4f, anti-Ly6c and anti-S100A9 quantifications were expressed as positive area per HPF.

TUNEL assay in pancreatic sections. Cell death was analysed in pancreatic sections from mice receiving chronic treatment with sEV by using the DeadEnd™ Fluorometric TUNEL System (G3250, Promega, Madison, WI, USA) according to supplier's instructions. Briefly, tissue sections (5 µm) were de-waxed by two washes with xylene and rehydrated through a descending series of ethanol dilutions (100, 95, 85, 75, 50%) and washed with 0.85% NaCl. The slides were then fixed 15 min with 4% PFA before permeabilization with Proteinase K (20 µg/ml for 10 min), after which the samples were fixed again for 5 min with 4% PFA before 10 min incubation in equilibration buffer (EB). The dead cells were then labelled with a mix of 1:10 nucleotides and 1:50 of Terminal deoxynucleotidyl transferase (Tdt) enzyme in EB for 60 min at 37 °C, avoiding light exposure and covered with a plastic slip. The reaction was stopped by washing the preparations in 2x saline-sodium citrate buffer. Nuclei were stained for 5 min with DAPI in PBS (1:1000) and sections were mounted in Fluoromount-G Mounting Medium (00-4958-02, Invitrogen, Waltham, MA, USA) and images were acquired with an LSM710 confocal microscope (Carl Zeiss, Oberkochen, Germany).

Antibodies for immunostaining procedures are listed in ESM Table 2.

Reverse transcription real-time quantitative PCR. Total RNA from tissues or cells was extracted using TRIzol Reagent (15596026, Thermo Fisher Scientific, Waltham, MA, USA). Total RNA from pancreatic islets (50 islets) was extracted using the RNeasy Micro Kit (74004, Qiagen, Venlo,

Netherlands) as recommended by manufacturer with minor modifications. Beta-mercaptoethanol was added to RLT buffer to decrease RNAse activity and mechanical shearing was performed by passing the lysate 5-10 times through a 29-gauge needle. Reverse transcription was performed using the High-Capacity cDNA Reverse Transcription Kit with random primers and MultiScribe™ III Reverse enzyme (4368814, ThermoFisher Scientific, Waltham, MA, USA). Quantitative real-time polymerase chain reaction was performed in a 7900 HT-Fast real-time PCR (Life Technologies) with TaqMan Universal PCR Master Mix or SYBR Green detection protocol. Reverse transcription and RT-qPCR was carried out at the Genomics Unit of IIBm (CSIC/UAM, Madrid, Spain) using 5 or 50 ng of cDNA depending on gene expression. The relative changes in gene expression were calculated using the $2^{-\Delta\Delta Ct}$ (cycle threshold) quantification method, normalized to expression levels of the TBP or GAPDH. Additionally, RT-PCR was used for the detection of XBP1 splicing, using DNA AmpliTools Green Master Mix (10432, Biotools, San Francisco, CA, USA).

Primer sequences and Taqman probes used are shown in ESM Table 3.

Protein extracts preparation and Western Blot. To obtain total cell lysates, attached cells were scrapped off and incubated for 10 min on ice with lysis buffer (10 mM Tris pH 7.5, 5 mM EDTA, 50 mM NaCl, 30 μM sodium pyrophosphate, 50 mM sodium fluoride (NaF, S7920, Sigma-Aldrich, San Louis, M, USA), 100 µM o-vanadate sodium (S6508, Sigma-Aldrich), 1% Triton X-100, 1 mM phenylmethylsulfonyl fluoride (PMSF, P7626, Sigma-Aldrich, San Louis, MO, USA), and 10 μg/ml protease inhibitors (P8340, Sigma-Aldrich, San Louis, MO, USA), pH 7.4-7.6. Protein content was determined with Bradford reagent (500-0006, Bio-Rad, Hercules, CA, USA) or BCA assay (23227, Thermo Fisher Scientific, Waltham, MA, USA). For tissue liver samples, a portion was lysed in lysis buffer (50 mM HEPES pH 7.5, 1 % Triton X-100, 50 mM $Na_4P_2O_7$ (P8010, Sigma-Aldrich, San Louis, MO, USA), 100 mM NaF, 10 mM EDTA, 10 nM Na_3VO_4, 1 mM PMSF and 10 μg/ml protease inhibitors, using a tissue homogenizer. After centrifugation at 100,000 × g for 40 min at 4 °C, the supernatant was again centrifuged at 100,000 × g for 40 min at 4 ºC and the resulting supernatant was collected. Protein levels were quantified using the BCA assay. Protein samples were boiled at 95 ºC for 5 min in loading buffer (100 mM Tris pH 6.8, 10% glycerol, 4% sodium dodecyl sulphate (SDS), 0.2% bromophenol blue and 2 mM β-mercaptoethanol and submitted to 8-15% SDS-PAGE. Proteins were transferred to polyvinylidene difluoride (IPVH00010, Merck Millipore, Burlington, MA, USA) membranes, and, after blocking with 4% BSA or 5% non-fat dry milk, membranes were incubated o/n with primary antibodies. Immunoreactive bands were visualized with ECL chemiluminescent substrate (170-5061, Bio-Rad, Hercules, CA, USA) developed in a ChemiDoc imager (733BR-3548, Bio-Rad, Hercules, CA, USA). Densitometric analysis of the bands was performed using ImageJ 2.0 Software (NIH). The following proteins were analysed: p-JNK, JNK, IκBα, vinculin, p-eIF2α, eIF2α, p33 active caspase-1, tubulin, pAkt473, Akt, CD63, CD81, TSG101, PCNA, GRP78 and Fetuin A.

Antibodies for Western blot are listed in ESM Table 4.

ESM TABLES

ESM Table 1. Antibodies used in flow cytometry.

Antibody	Supplier	Cat. no	Host specie	Dilution
Anti-CD45 BV570	BioLegend	103136	Rat	1:100
Anti-F4/80 APC	eBioscience	17-4801-82	Rat	1:100
Anti-CD11b Alexa 700	eBioscience	56-0112-82	Rat	1:100
Anti-Ly6c FITC	BioLegend	128006	Rat	1:100
Anti-Ly6g PE	Pharmingen	551461	Rat	1:100
Anti-CD206 PE	BioLegend	141706	Rat	1:100
Anti-CD3 PECY7	BioLegend	100320	Armenian Hamster	1:100
Anti-CD4 eF450	eBioscience	48-0042-82	Rat	1:100
Anti-CD8 APC	BioLegend	100712	Rat	1:100

ESM Table 2. Antibodies used in immunostaining assays.

Antibody	Supplier	Cat. no	Host specie	Dilution
Anti-Clec4f	R&D Systems	AF2784	Goat	1:50
Anti-F4/80	Provided by A.Castrillo (IIBm, Madrid)		Rat	1:50
Anti-Ki67	Abcam	ab16667	Rabbit	1:100
Anti-Ly6c	Provided by A.Castrillo (IIBm, Madrid)		Rat	1:50
Anti-p65 NF-κB	Cell Signaling Technology	8242	Rabbit	1:100
Anti-Pdx1	DSHB	F109-D12	Rat	1:100
Anti-αSMA	Invitrogen	14-9760-82	Mouse	1:100
Anti-S100A9	Abcam	ab242945	Rabbit	1:500
Anti-glucagon	Sigma-Aldrich	G2654	Mouse	1:300
Anti-insulin	Invitrogen	PA126938	Guinea pig	1:500

ESM Table 3. Primers and Taqman probes used in RT-qPCR.

Gene	Specie	Forward (5′ → 3′)	Reverse (5′ → 3′)
Proinflammatory cytokines genes			
Adgre1	Mouse	CTTTGGCTATGGGCTTCCAGTC	GCAAGGAGGAGGACAGAGTTTATCGTG
Ccl2	Mouse	AAAAACCTGGATCGGAACCAA	CGGGTCAACTTCACATTCAAAG
Ccl3	Mouse	TTCTCTGTACCATGACACTCTGC	CGTGGAATCTTCCGGCTGTAG
Ifng	Mouse	ACAATGAACGCTACACACTGC	GGATTTTCATGTCACCATCCTTT
Il1b	Mouse	CTGGTGTGTGTGACGTTCCCATTA	CCGACAGCACGAGGCTTT
	Rat	CACCTCTAAGCAGAGCACAG	GGGTTCCATGGTGAAGTCAAC
Il1r	Mouse	GTCACGAACCAAACCTGTGC	CAGTTCACTGCCTCGACTGT
Il6	Mouse	CCTACCCCAATTTCCAATGCT	TATTTTCTGACCACAGTGAGGAATG
Tlr4	Mouse	CCTCTGCCTTCACTACAGAGACTTT	TGTGGAAGCCTTCCTGGATG
Tnf	Mouse	CCCTCACACTCAGATCATCTTCT	GCTACGACGTGGGCTACAG
Profibrotic genes			
Acta2	Mouse	CCCAGAVATCAGGGAGTAATGG	TCTATCGGATACTTCAGCGTCA
ER stress genes			
Chop	Rat	AAGCAGAAACCGGTCCAATT	TTCTTCCTCTTCGTTTCCTG
Xbp1	Rat	ACACGCTTGGGGATGGATGC	CCATGGGAAGATGTTCTGGG
β-cell genes			
Ins1	Mouse	TAGTGACCAGCTATAATCAGA	AACGCCAAGGTCTGAAGGTCC
	Rat	TCTTCTACACACCCAAGTCCCG	AGTGCCAAGGTCTGAAGATCCC
Ins2	Mouse	CCCTGCTGGCCCTGCTCTT	GGTCTGAAGGTCACCTGCT
	Rat	ATGGCCCTGTGGATCCGCTT	CTAGTTGCAGTAGTTCTCCA
Pdx1	Mouse	GAACCCGAGGAAAACAAGAGG	GTTCAACATCACTGCCAGCTC
	Rat	GGTGCCAGAGTTCAGTGCT	GGCACTTCGTATGGGGAGAT
Gck	Mouse	TGCCCTCCTCTGATTCGATG	GCAGGCTGACACCCAACTG
Housekeeping genes			
Actb	Rat	AGGAGGAGCAATGATCTTGATCTT	TCCTTCCTGGGCATGGAG
Gapdh	Rat	ACAGCAACAGGGTGGTGGAC	TTTGAGGGTGCAGCGAACTT
Tbp	Mouse	GGAGAATCATGGACCAGAACA	GATGGGAATTCCAGGAGTCA
Taqman probes			
Gene	Specie	Supplier	Cat. no
Gck	Mouse	Applied Biosystems, CA, USA	Mm00439129_m1Gck
Tbp	Mouse	Applied Biosystems, CA, USA	Mm00446973_m1

ESM Table 4. Antibodies used in Western blot.

Antibody	Supplier	Cat. no	Host specie	Dilution
Anti-Akt	Cell Signaling Technology	4691	Rabbit	1:10000
Anti-cleaved Caspase 1	Santa Cruz	sc-514	Rabbit	1:1000
Anti-cleaved Caspase 3 (Asp-175)	Cell Signaling Technology	9661	Rabbit	1:1000
Anti-CD63	Santa Cruz	sc-5275	Mouse	1:1000
Anti-CD81	Santa Cruz	sc-166029	Mouse	1:1000
Anti-eIF2α	Cell Signaling Technology	9722	Rabbit	1:1000
Anti-Fetuin A	Santa Cruz	sc-166531	Mouse	1:1000
Anti-GRP78	Santa Cruz	sc-13968	Rabbit	1:1000
Anti-IκBα	Santa Cruz	sc-371	Rabbit	1:1000
Anti-JNK	Santa Cruz	sc-571	Rabbit	1:1000
Anti-mouse	Santa Cruz	sc-516102	Goat	1:10000
Anti-PCNA	Santa Cruz	sc-56	Mouse	1:1000
Anti-phospho-Akt	Cell Signaling Technology	4058	Rabbit	1:1000
Anti-phospho-eIF2α	Cell Signaling Technology	9721	Rabbit	1:1000
Anti-phospho-JNK	Cell Signaling Technology	9251	Rabbit	1:1000
Anti-rabbit	Bethyl Laboratories	A120-108P	Goat	1:20000
Anti-TSG101	Abcam	ab30871	Rabbit	1:1000
Anti-tubulin	Sigma Aldrich	T5168	Mouse	1:5000
Anti-vinculin	Santa Cruz	sc-73614	Mouse	1:20000

ESM FIGURES

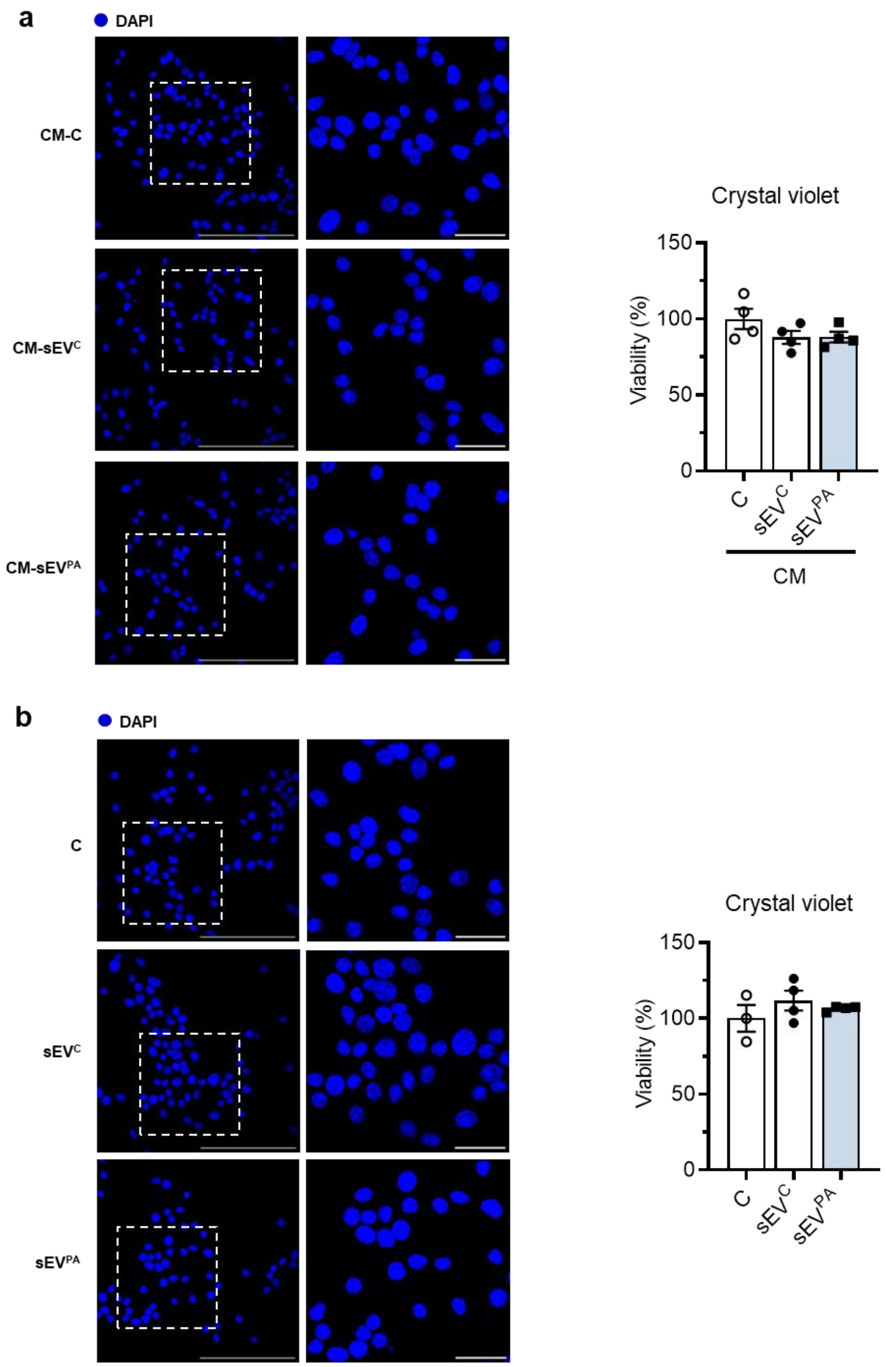

ESM Figure 1. Viability of INS-1 cells after stimulation with conditioned medium (CM) from macrophages treated with sEV or after stimulation with sEV directly. (a) Analysis of the presence of apoptotic nuclei by DAPI staining (left panel) and quantification of the percentage of cellular viability by crystal violet staining (right panel) in INS-1 beta cells after stimulation with the CM from macrophages treated with sEV (CM-sEV^{C}, CM-sEV^{PA}) compared to INS-1 cells receiving CM from non-treated macrophages (CM-C) (scale bars, 100 µm and 25 µm). **(b)** Analysis of the presence of apoptotic nuclei by DAPI staining (left panel) and quantification of the percentage of cellular viability by crystal violet staining (right panel) in INS-1 cells after stimulation with sEV (sEV^{C}, sEV^{PA}) compared to non-treated cells (C), (scale bars, 100 µm and 25 µm).

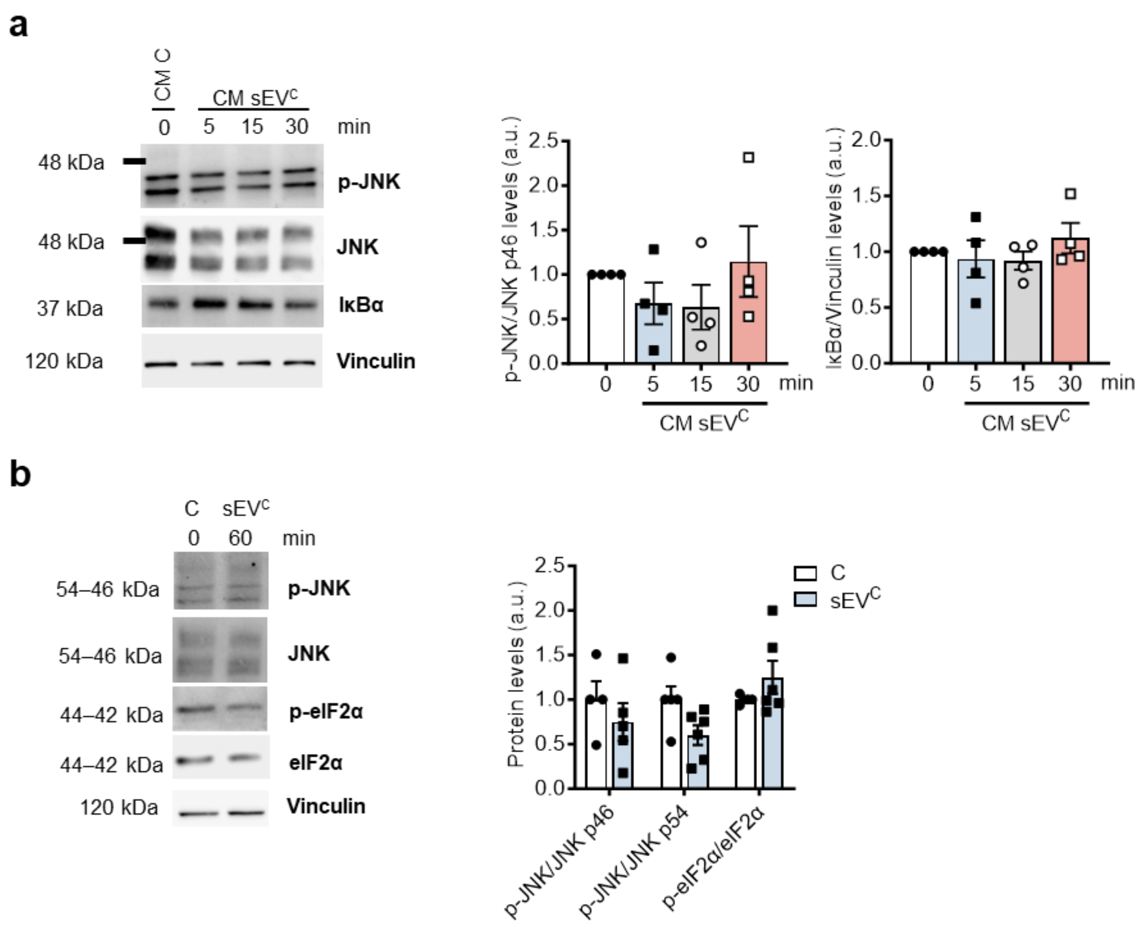

ESM Figure 2. (a) INS-1 beta cells do not respond to CM-sEV^{C} in inducing JNK phosphorylation or IκBα degradation. Representative Western blot analysis with the indicated antibodies: p-JNK/JNK (p46) (n=4/group) and IκBα (n=4/group) and quantification. **(b) INS-1 beta cells do not respond to sEV^{C} in inducing JNK and eIF2α phosphorylation.** Representative western blot analysis with the indicated antibodies, p-JNK/JNK and p-eIF2α/eIF2α and quantification (n=4-6/group). a.u., arbitrary units.

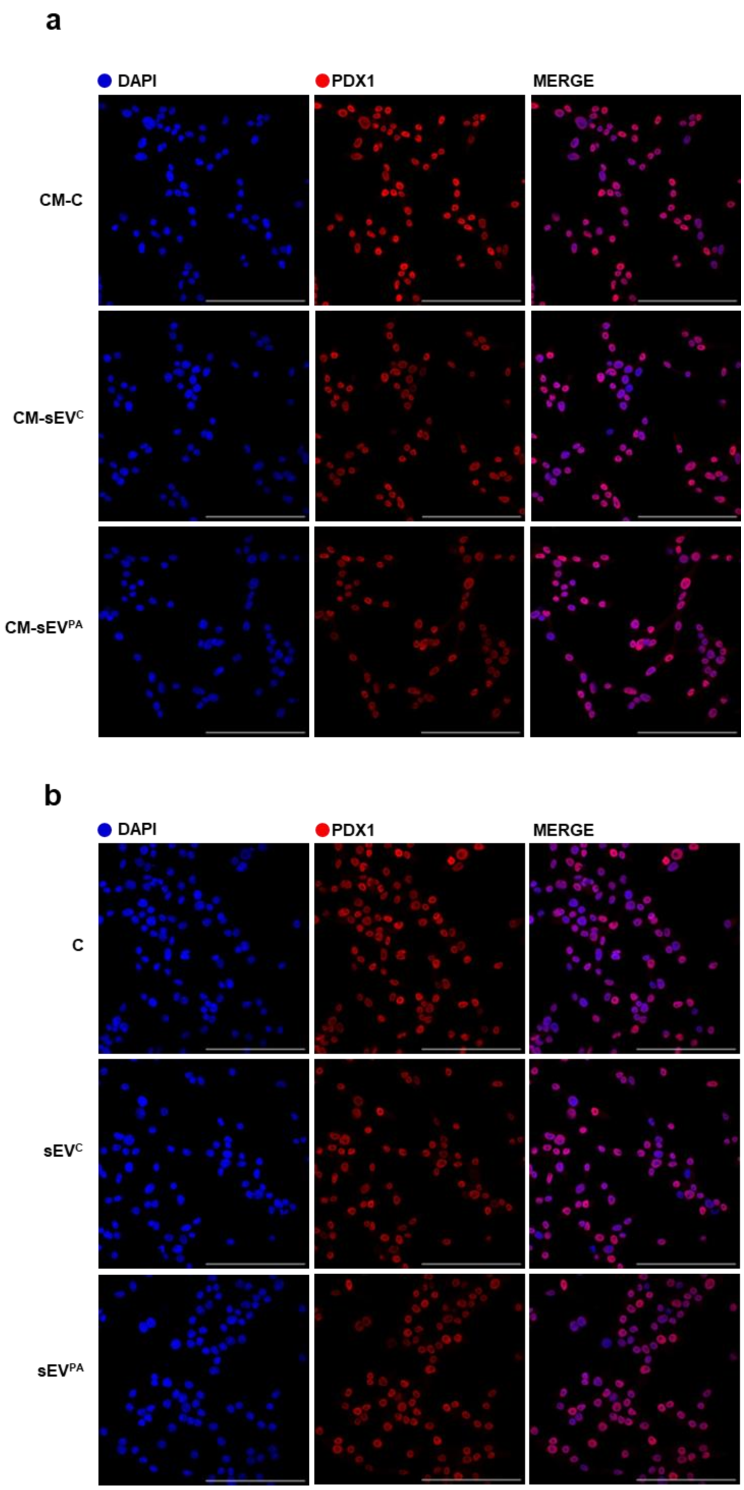

ESM Figure 3. Analysis of PDX1 translocation in INS-1 cells after stimulation with conditioned medium (CM) from macrophages treated with sEV or after direct treatment with sEV. (a) Representative PDX1 immunofluorescence images of INS-1 cells after stimulation with CM from non-treated macrophages (CM-C) or macrophages treated with sEV (CM-sEV^{C}, CM-sEV^{PA}), (scale bar, 100 µm). **(b)** Representative PDX1 immunofluorescence images of non-treated INS-1 cells (C) and after stimulation with sEV (sEV^{C}, sEV^{PA}), (scale bar, 100 µm).

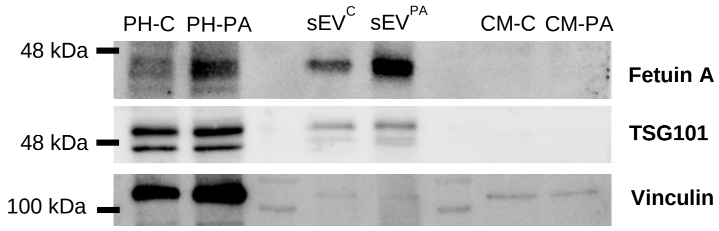

ESM Figure 4. Representative Western blot of Fetuin A in secretory primary hepatocytes (PH), sEV and the conditioned medium (CM) from macrophages after 100,000 × g ultracentrifugation. Vinculin was used as loading control. 40 µg of protein was loaded for each condition (n=pool from 3 mice).

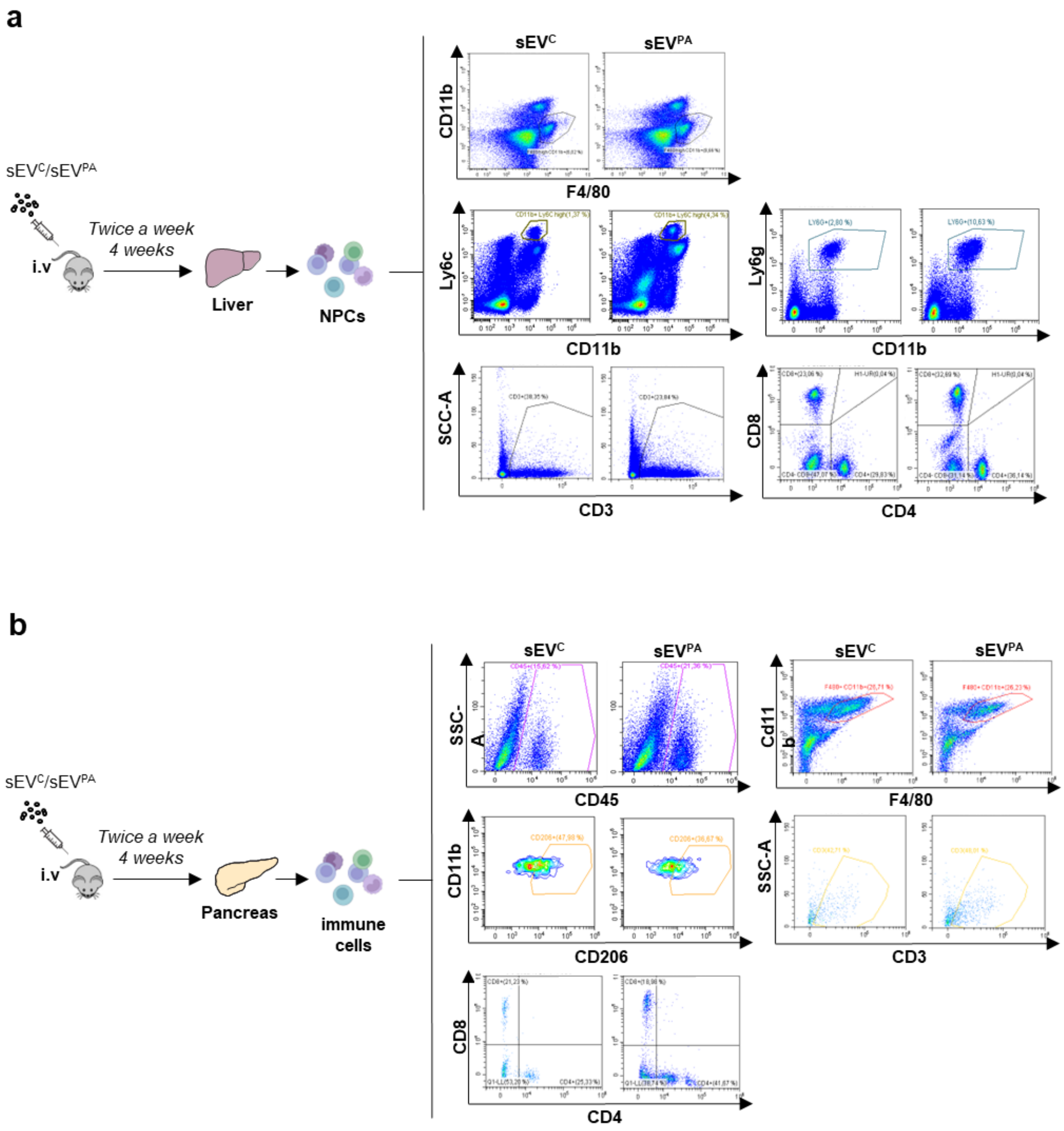

ESM Figure 5. (a) Flow cytometry gating strategy used to immunophenotype liver non-parenchymal cells (NPCs) and **(b)** pancreatic immune cells.

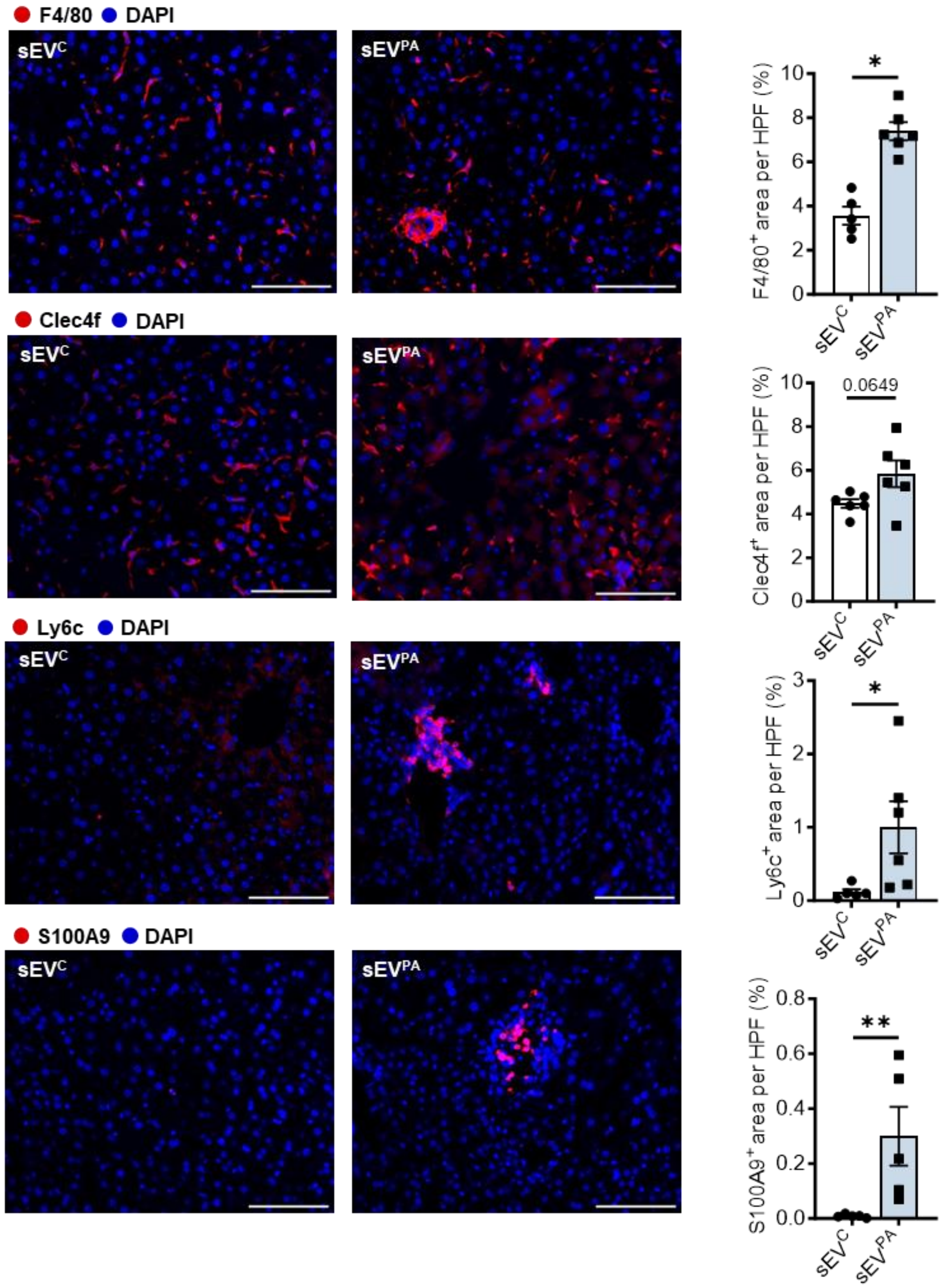

ESM Figure 6. Effects of chronic Hep-sEV administration on hepatic immune cells. Representative images of liver immunofluorescence for F4/80 (sEV^{C} n=5, sEV^{PA} n=6), Clec4f (n=6/group), Ly6c (sEV^{C} n=5, sEV^{PA} n=6) and S100A9 (n=6/group) (scale bar, 100 µm) and positive area quantification per high-powered field (HPF) (20X). Data are expressed as the mean ± S.E.M. * P<0.05, ** P<0.01, compared to sEV^{C}, Mann-Whitney U test.

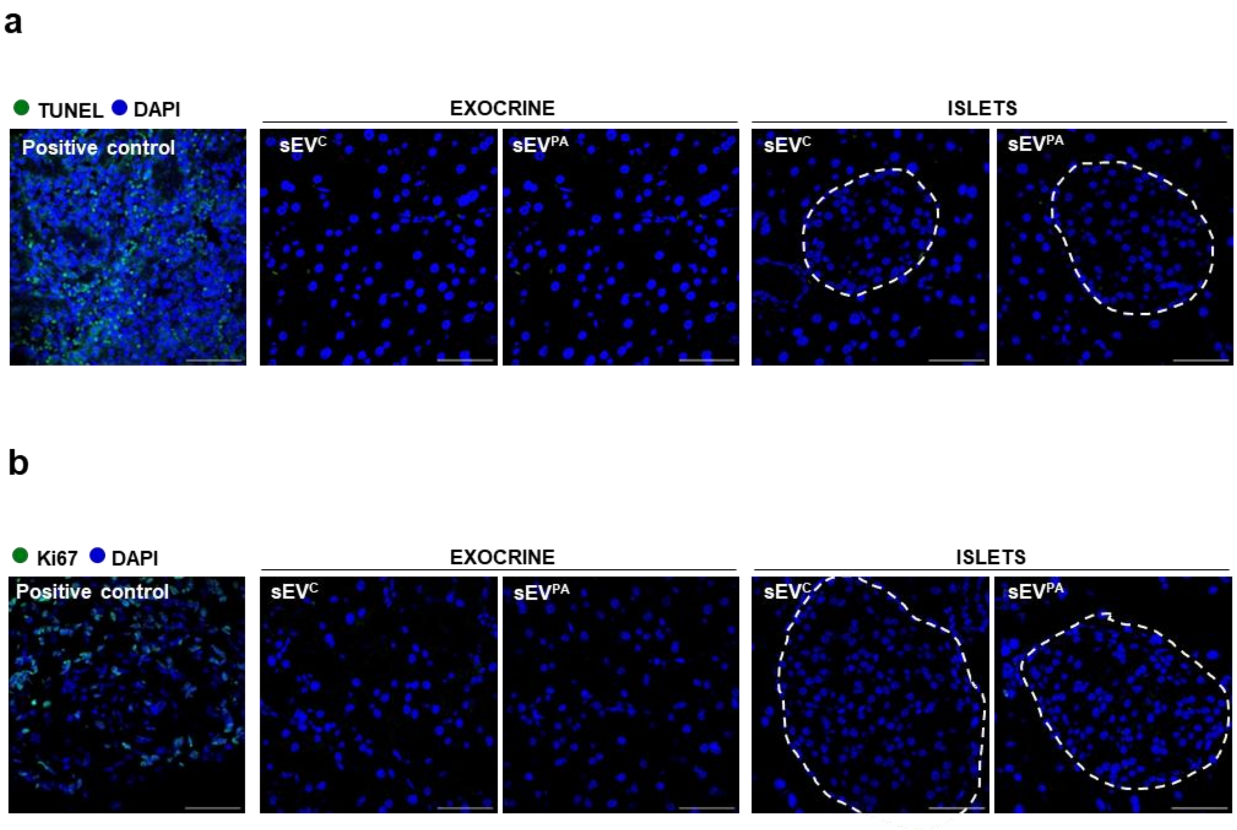

ESM Figure 7. Apoptosis and proliferation analysis in pancreatic sections from mice after chronic treatment with sEV^{C} or sEV^{PA}. (a) Representative images of TUNEL assay in pancreatic sections (exocrine and islets). **(b)** Representative images of Ki67 immunostaining in pancreatic sections (exocrine and islets). In a, b: Mouse pancreatic ductal adenocarcinoma sections were used as positive control.

Checklist for reporting human islet preparations used in research

Adapted from Hart NJ, Powers AC (2018) Progress, challenges, and suggestions for using human islets to understand islet biology and human diabetes. Diabetologia https://doi.org/10.1007/s00125-018-4772-2

**Islet preparation**	**1**	**2**	**3**	**4**	**5**	**6**	**7**	**8[a]**
**MANDATORY INFORMATION**								
Unique identifier	HP-24151	HP-24261-01	HP-24346-01					
Donor age (years)	38	63	39					
Donor sex (M/F)	M	M	F					
Donor BMI (kg/m^2)	23.2	19.5	28.2					
Donor HbA$_{1c}$ or other measure of blood glucose control	5.7%	5.5%	5.4%					
Origin/source of islets[b]	Tebu bio	Tebu bio	Tebu bio					
Islet isolation centre	Prodo Laboratories, Inc	Prodo Laboratories, Inc	Prodo Laboratories, Inc					
Donor history of diabetes? Please select yes/no from drop down list	No	No	No					
**If Yes, complete the next two lines if this information is available**								
Diabetes duration (years)								
Glucose-lowering therapy at time of death[c]								
**RECOMMENDED INFORMATION**								
Donor cause of death	Anoxic event	Head trauma	Anoxic event					
Warm ischaemia time (h)								
Cold ischaemia time (h)								
Estimated purity (%)	90	90	90					
Estimated viability (%)	95	95	95					
Total culture time (h)[d]								
Glucose-stimulated insulin secretion or other functional measurement[e]	GSIS, secreted perfectly	GSIS, secreted perfectly	GSIS, secreted perfectly					
Handpicked to purity? Please select yes/no from drop down list	Yes	Yes	Yes					
Additional notes								

[a]If you have used more than eight islet preparations, please complete additional forms as necessary
[b]For example, IIDP, ECIT, Alberta IsletCore
[c]Please specify the therapy/therapies
[d]Time of islet culture at the isolation centre, during shipment and at the receiving laboratory
[e]Please specify the test and the results
